# Genome-wide functional analyses of plant coiled–coil NLR-type pathogen receptors reveal essential roles of their N-terminal domain in oligomerization, networking, and immunity

**DOI:** 10.1371/journal.pbio.2005821

**Published:** 2018-12-12

**Authors:** Tadeusz Wróblewski, Laurentiu Spiridon, Eliza Cristina Martin, Andrei-Jose Petrescu, Keri Cavanaugh, Maria José Truco, Huaqin Xu, Dariusz Gozdowski, Krzysztof Pawłowski, Richard W. Michelmore, Frank L.W. Takken

**Affiliations:** 1 The Genome Center, University of California–Davis, Davis, California, United States of America; 2 Department of Bioinformatics and Structural Biochemistry, Institute of Biochemistry of the Romanian Academy, Bucharest, Romania; 3 Department of Experimental Design and Bioinformatics, Faculty of Agriculture and Biology, Warsaw University of Life Sciences, Warsaw, Poland; 4 Departments of Plant Sciences, Molecular & Cellular Biology, and Medical Microbiology & Immunology, University of California–Davis, Davis, California, United States of America; 5 Department of Medical Microbiology and Immunology, University of California–Davis, Davis, California, United States of America; 6 Molecular Plant Pathology, Swammerdam Institute for Life Sciences, University of Amsterdam, Amsterdam, the Netherlands; The Sainsbury Laboratory, United Kingdom of Great Britain and Northern Ireland

## Abstract

The ability to induce a defense response after pathogen attack is a critical feature of the immune system of any organism. Nucleotide-binding leucine-rich repeat receptors (NLRs) are key players in this process and perceive the occurrence of nonself-activities or foreign molecules. In plants, coevolution with a variety of pests and pathogens has resulted in repertoires of several hundred diverse NLRs in single individuals and many more in populations as a whole. However, the mechanism by which defense signaling is triggered by these NLRs in plants is poorly understood. Here, we show that upon pathogen perception, NLRs use their N-terminal domains to transactivate other receptors. Their N-terminal domains homo- and heterodimerize, suggesting that plant NLRs oligomerize upon activation, similar to the vertebrate NLRs; however, consistent with their large number in plants, the complexes are highly heterometric. Also, in contrast to metazoan NLRs, the N-terminus, rather than their centrally located nucleotide-binding (NB) domain, can mediate initial partner selection. The highly redundant network of NLR interactions in plants is proposed to provide resilience to perturbation by pathogens.

## Author summary

The ability to induce defenses in response to pathogen attack is a critical feature of immunity in any organism. Nucleotide-binding leucine-rich repeat receptors (NLRs) are key players in this process and have evolved to perceive the occurrence of nonself-activities or foreign molecules. In plants, coevolution with a variety of pests and pathogens has resulted in repertoires of several hundred diverse NLRs in single individuals and many more in populations as a whole. The mechanism by which defense signaling is triggered by these NLRs is poorly understood. Here, we show that upon pathogen perception, NLRs use their N-terminal domains to transactivate other receptors. Their N-terminal domains homo- and heterodimerize, suggesting that plant NLRs oligomerize upon activation, similar to the vertebrate NLRs; however, consistent with their large number in plants, their complexes are highly heterometric. Also, in contrast to metazoan NLRs, their N-terminus, rather than their centrally located nucleotide-binding (NB) domain, can mediate initial partner selection. The highly redundant network of NLR interactions is proposed to provide resilience to perturbation by pathogens.

## Introduction

Signal Transduction ATPases (STAND proteins) comprise an ancient group of modular proteins sharing a conserved nucleotide-binding (NB) domain [1]. STAND proteins are present in *Archaea*, *Bacteria*, and *Eukaryota*, implying a common, ancient evolutionary origin [1,2]. Duplications and associations of the NB domain with other functional domains have driven their divergent evolution, allowing them to participate in multiple signaling processes. Typically, STAND proteins act as intracellular receptors triggering cellular signaling responses upon elicitation. In animals, members of two major groups of STAND proteins, the nucleotide-binding oligomerization domain (NOD)-like receptors (also referred to as NACHT [1] or animal nucleotide-binding leucine-rich repeat receptors [NLR]) and the nucleotide-binding ARC [1] domain (NB–ARC or simply NB)-containing apoptotic proteins, include some of the key players involved in the induction of immune responses or programmed cell death (pcd), respectively [1,3].

The NB domain controls the transition from a resting to an activated state through its involvement in differential adenosine diphosphate (ADP)/ATP (or guanosine triphosphate [GTP]) binding and nucleotide hydrolysis [1,4]. The best studied STAND protein, the Apoptotic Protease Activating Factor 1 (APAF1), induces pcd in human cells upon perception of cytochrome C released from mitochondria [5]. APAF1 activation triggers a conformational change that frees its C-terminal caspase-recruitment domain (CARD) and exposes its NB domain, enabling interactions with other APAF1 monomers [5]. Subsequently, intermolecular interactions are formed between the NB domains of adjacent monomers allowing formation of a circular heptamer called the apoptosome. The apoptosome is the active form of the protein and can initiate a caspase-signaling cascade resulting in pcd [6,7]. Similarly, APAF1 orthologs in Drosophila and *Caenorhabditis elegans* (DARK1 and CED-4, respectively) form multimeric assemblies upon their activation and trigger pcd [8,9]. The NOD domains of metazoan NOD-like receptors also interact to form oligomeric assemblies of nine or more subunits [10,11]. The bacterial transcription factor MalT, which is evolutionarily related to ancestral STAND proteins, similarly oligomerizes to form a curved homopolymer upon its activation [12]. In all of these cases, oligomerization of the central NB or NOD domain serves to bring the N-terminal domains in close proximity, allowing their partners to interact and induce downstream signaling [13]. Hence, formation of apoptosome-like complexes facilitating the induced proximity of N-terminal domains may represent a common feature of STAND proteins [13].

In plants, numerous STAND receptors are present, and those that have been functionally characterized are mostly involved in innate immunity, conferring protection against diverse pests and pathogens [14,15]. At least part of their recognition specificity can be attributed to highly variable leucine-rich repeats (LRRs), defining the C-terminal portion of plant NB-LRR receptors or plant NLRs. The majority of hundreds of genetically characterized disease resistance traits in plants map to genes encoding NLRs; the large numbers of such sequences in the genome and their high diversity reflect dynamic interactions between hosts and rapidly evolving pathogens [16]. NLRs are integral to effector-triggered immunity (ETI) [14] through direct or indirect recognition of effectors (virulence-enhancing proteins secreted by pathogens during infection). ETI complements the less specific microbe-associated molecular pattern (MAMP)-triggered immunity (MTI) mediated by extracellular receptor-like kinases (RLKs) [14].

The NB–ARC domains of APAF1 (a NB–ARC-WD40 type of receptor) and its orthologs are the most similar at the sequence level of the NLRs outside the plant kingdom. Instead of a CARD, the N-termini of plant NLRs contain (with some exceptions) sequences similar to either Toll/interleukin-1 receptor (TIR) or a coiled–coil (CC) domains, allowing subclassification into TIR–NLRs (TNLs) and CC–NLRs (CNLs) [17]. In dicotyledonous plants, TNLs are sometimes more abundant than CNLs; however, in monocots, CNLs provide the core repertoire of receptors mediating ETI [18]. NLR activation most likely releases the N-terminal CC or TIR domain (similarly to the release of CARD upon APAF1 activation) to induce defense responses, which are often concomitant with pcd [19]. This type of ETI response is often referred to as the hypersensitive response (HR) [20]. Even though plant NLRs trigger pcd, universal signaling mediators (such as caspases in case of APAF1) activated by plant NLRs have not been identified to date [3]. Unlike in vertebrates, which typically have fewer than two dozen NB–ARC or NOD proteins encoded in their genomes, the number of NLRs expressed in a single plant may exceed several hundred [21]. This large number provides potential for homo/heteromerization, but formation of multimeric complexes in plants following NLR activation has so far not been conclusively demonstrated [22,23]. Hence, it was currently an open question whether plant STAND proteins oligomerize upon activation.

Consistent with their involvement in downstream signaling, in planta expression of TIR or CC domains alone can induce HR [24–27], but their potential role in NLR oligomerization is unclear. Some TIR domains form dimers or even homotypic heteromultimers (implying the existence of higher-order complexes) when expressed without the adjacent NB and LRR domains, suggesting their involvement in NLR multimerization [23,28–30]. The extended CC domain of the barley powdery mildew resistance 10 (Mla10) receptor (Mla10-CC) forms a helix-loop-helix rod-shaped homodimer, and mutations affecting dimerization compromise Mla-mediated resistance against powdery mildew [26,31]. Subsequent structural studies proposed that only the extended Mla10-CC dimerizes and folds into a monomeric four-helix bundle structure, a structure similar to that reported for CCs of wheat stem rust resistance 33 (Sr33) and potato virus X resistance (Rx) receptors [32,33]. Full-length CC domains of Mla10 and Sr33 form homomeric and heteromeric associations, disruption of which compromises induction of cell death [32,34]. The observed monomeric and dimeric CC structures may reflect different states in a receptor’s activation [35]. Similarly, self-association of CC corresponding to Arabidopsis resistance to *Pseudomonas syringae* (avrRpm1) (RPM1) receptor appeared to be required for its activity [36]. Furthermore, heterodimer formation between the CC domains of two rice CNLs, RGA4 and RGA5, is required to respond to the Avr-Pia effector from the fungus *Magnaporthe oryzae* [22]. So although homo- and heterodimerization of N-terminal domains had been shown for some CNL proteins, it was unknown whether the full-length proteins form higher-order complexes in plants and, if so, what the role of the CC was in this process [37].

NLRs in plants can be categorized into two functional groups, the sensors and the actors (also referred to as helpers or activators), in which sensor NLRs are proposed to detect the pathogen-derived effectors, and an evolutionary conserved downstream-signaling partner NLR triggers defense [38–40]. The sensor/actor concept emerged after the discovery of a conserved class of CNLs referred to as CC_R_–CNLs, RPW8-, or NRG-like CNLs that share a distinct consensus sequence of their CC (CC_R_) domains [24,38]. CC_R_–CNLs are required for the activity of some canonical CNLs and TNLs, and expression of the CC_R_ domain alone triggers extensive pcd, consistent with their proposed actor role in downstream immune–signaling [24,39]. Physical association between putative sensors and CC_R_–CNLs had not been demonstrated and how the phylogenetic diversity of CC domains reflects their roles in CNL cross-activation and signaling remains unknown [37].

To investigate the network of CNLs mediating ETI in plants and to elucidate the role(s) of CC domains in induction defense, we performed extensive genome-wide functional and in silico analyses of N-termini containing a predicted CC domain of nearly all of the CNLs in *Arabidopsis thaliana* ecotype Columbia-0 (*At*-Col-0). By combining data on their sequence variation with their ability to homo-/heterodimerize and induce cell death and/or disease resistance in three different plant species, we identified regions required for their function. Subsequent genetic mapping and reverse complementation confirmed the involvement of canonical receptors in CNL signaling in other plant species, implying that the role of CC domains in downstream signaling involves transactivation of other CNLs. Surprisingly, a highly variable part of the CC domain is required for this transactivation. Accordingly, we present two lines of evidence that NLR receptors form a network mediated by physical and functional associations.

## Results

### Structure-based alignment of N-terminal domains of CNL receptors in *At*-Col-0 discloses four major groups

CC domains are defined by heptad repeats of hydrophobic residues (L, I, or V) [41]; these form a binding interface of α-helical secondary structure that is involved in helix-to-helix binding [42]. We aligned the sequences of N-terminal fragments representing all CNL receptors predicted in *At*-Col-0, including sequences encoding truncated receptor proteins referred to as CC–NBs (CNs) [17]. The alignment was refined using structure-based words (patterns such as hydrophobic heptad repeats along with their predicted accessibility) devised from crystallographic/NMR data of Mla10-CC, Sr33-CC, and Rx-CC [26,32,33]. The alignment (S1 Fig) revealed four major Groups that we designated Group A, B, C, and D and an E outgroup (S2 Fig). The naming of these Groups is based on a previous study in which *At*-Col-0 CNLs were clustered based on the topology of their NB–ARC domains and the intron/exon features of the encoding genes [17]. A classical sequence alignment (Clustal Omega) [43] using the CC and NB–ARC sequences resulted in a very similar cladogram as compared to the NB–ARC domain sequence alone (S3 Fig), implying that the CC domains follow a similar pattern of diversification as the remaining part of the receptor. However, the obtained trees were not identical; whereas, for instance, Groups B and D are well-defined in both studies, three members of Group C (AT4G19060, AT5G45440, and AT5G45490) were placed with Group A in the CC–NB–ARC-based cladogram (S3 Fig). These differences can be attributed to high diversity and rather ambiguous alignments of the CC-containing fragment. However, for consistency with the existing literature, we applied the same letter designations as before [17].

**Fig 1:**
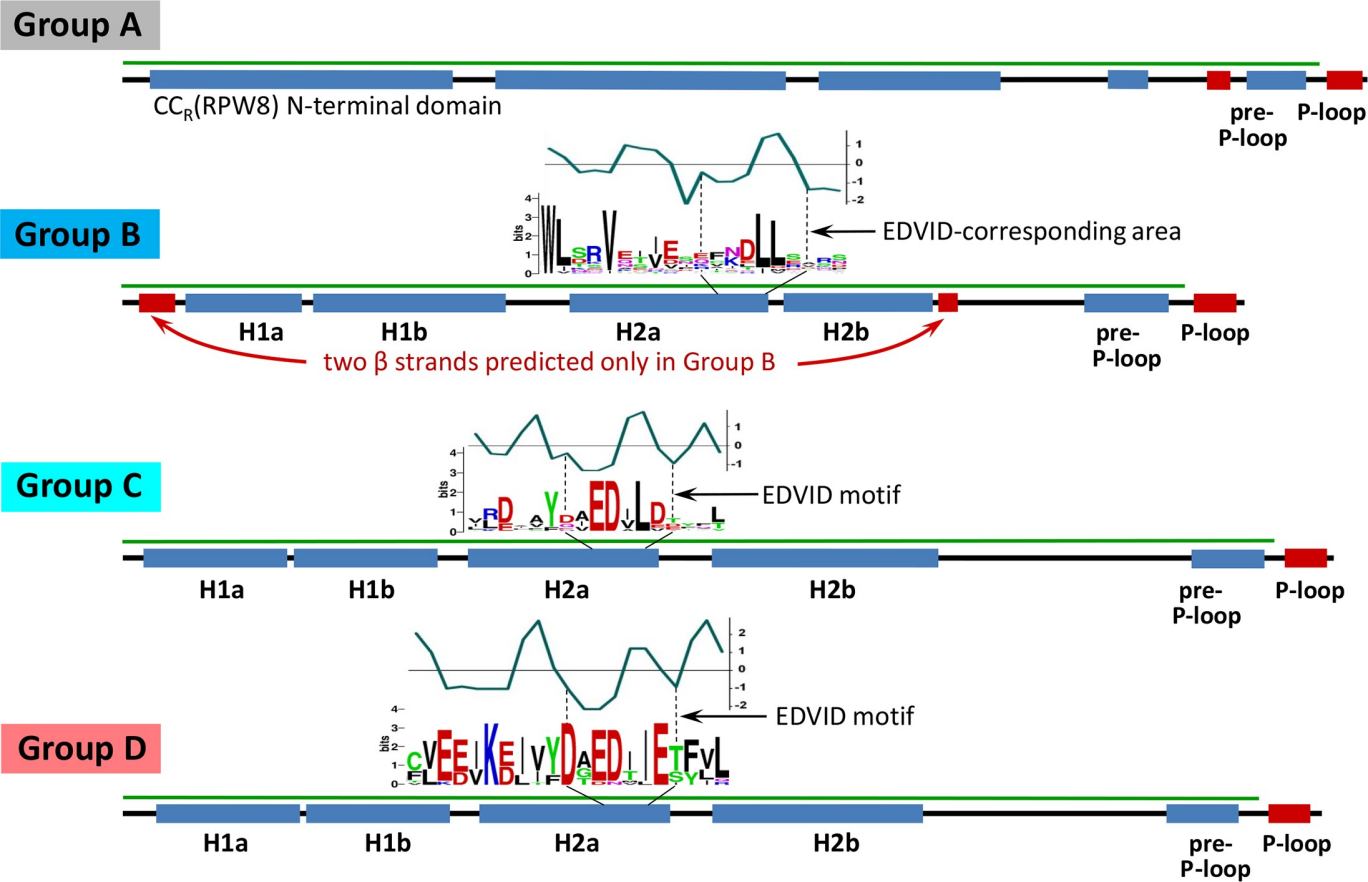
Schematic representation of the N-termini of four major Groups of CNL receptors discerned from the *At*-Col-0 genome. Group A, comprising six CC_R_(RPW8) CNL homologs, is different from the other Groups in regard to the distribution of the predicted α-helices (blue bars). Groups B through D share a similar organization, but only Groups C and D contain a conserved array of amino acids known as the EDVID motif [15,17]. Only Group B contains two predicted short N-terminal beta sheets (red bars) and predicted myristoylation and palmitoylation sites. The chart above each logo depicts the hydrophobicity pattern. The eight-amino acid–long fragments containing the EDVID motif in Groups C and D and EDVID-corresponding area in Group B show similar hydrophobicity profiles. Green lines define length of the ECCs. The colors highlighting the Groups are the same as used to aid visualization and comparisons to Figs 2 and 4, S2 Fig and S3 Fig. Alignments were created using PROMALS3D using default parameters [46]. *At*-Col-0, *Arabidopsis thaliana* ecotype Columbia-0; CC, coiled–coil; CNL, CC–NLR; ECC, extended CC domains.

**Fig 2:**
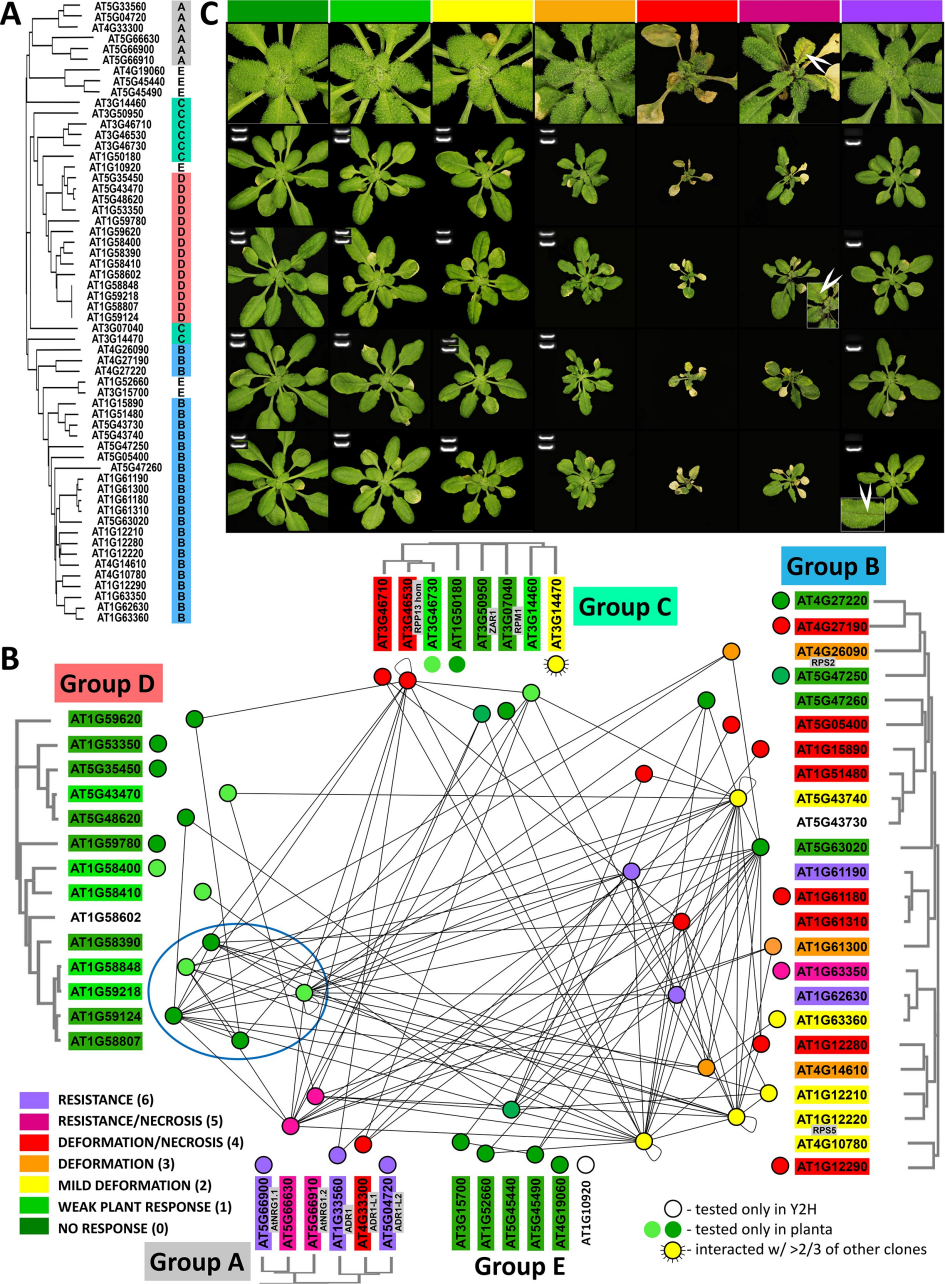
Phylogenetic relationship, interactome, and cell death induction by ECCs representing the CNL repertoire in *At*-Col-0. (A) Cladogram corresponding to the alignment of both CC and NB–ARC domains; Groups are labeled with letters and colors for better visualization. Group D forms a clade rooted in Group C. Members of Group E are juxtaposed among members of Groups B and C according to the alignment of the NB–ARC domain. (B) In the interactome, dots represent ECCs corresponding to CNLs clustered in Groups/clades and lines connecting dots show interactions. The color of each dot and the highlighting of the corresponding gene represents the phenotype of TRV–ECC infected as depicted in panel C. The blue oval encases five ECCs sharing four conserved amino acid residues (compare to Fig 3 and S1 Fig). (C) *At*-Col-0 plants infected with TRV carrying ECCs eight days post infection. In each column, four captures at the bottom show the typical plant reaction in each phenotypic category depicted by different color in the top row. The fifth, top capture shows a close up of the rosette’s center of a representative plant of each category. The same colors are used to visualize phenotypes induced by each ECC in panel B. White arrowheads point to necrosis developing along veins of infected plants. The categories of reactions were defined based on monitoring the magnitude of necrosis/resistance induction over many experiments following systemic TRV infection. Because resistant plants (purple) were phenotypically similar to plants showing moderate response (yellow and orange), PCR was used to determine the presence of the virus in newly emerging leaves as an indication of systemic TRV movement: a single white band indicates absence of the virus; two white bands indicate its presence. *At*-Col-0, *Arabidopsis thaliana* ecotype Columbia-0; CC, coiled–coil; CNL, CC–NLR; ECC, extended CC domain; NB–ARC; TRV, Tobacco Rattle Virus.

Group E gathered N-terminal fragments of the CN homologs lacking a clearly defined CC domain precluding their structure-based alignment (S1 Fig, S2 Fig). Group D had the highest average sequence identity (id.)/similarity (sim.) of the Groups to sequences of Mla10 or Sr33 (Mla10: id. 18.0%, sim. 44.3%; Sr33: id. 17.5%, sim. 39.2%; Rx: id. 11.1%, sim. 30.2%). Groups C and D, comprising eight and 14 members, respectively, were the most similar and shared the four predicted α helices of the CC domains of Mla10, Sr33, and Rx: H1a, H1b, H2a, and H2b [26,32] (Fig 1, S2 Fig). Even though all members of Group B also contain four predicted α helices, these helices did not align with helices predicted for Groups C and D (S2 Fig). Detailed secondary structural analysis of Groups C and D revealed a profile that resembled Mla10–CC and Sr33–CC more than Rx–CC (S1 File). This is seen especially in the H1a–H1b turn region; for these Groups, the separation of the first two α helices (H1a and H1b) was not consistently predicted, which is in agreement with crystallographic data for Mla10–CC, in which helices H1a and H1b form one single helix H1 [26]. However, the molecules may adopt different secondary structures in the presence of an interacting partner. In contrast, to the H1a–H1b region, the predicted helices H1b, H2a, and H2b were clearly separated from each other by areas of flexibility, which corresponds to turns in the three resolved CC structures [26,32,33] (Fig 1, S1 Fig, S2 Fig). The strongest sequence identity within Groups C and D occurs around the five-amino acid–long EDVID motif in helix H2a; in addition, Group D contains a conserved stretch of 10 polar amino acids immediately preceding this motif (S2 Fig). The CC–NB–ARC-based phylogeny (Fig 2A, S3 Fig) places Group D within Group C; thus, both can be considered a merged C/D Group. In accordance with earlier reports, we did not find a clearly distinguishable EDVID motif in Group A and Group B, yet in Group B the corresponding area of eight amino acids showed some conservation, including a hydrophobicity distribution similar to the EDVID motif (Fig 1, S1 Fig). In Group B, representing more than a third of all analyzed CNLs, structure-based alignments predicted the existence of two short β-strands immediately preceding the first H1a helix and immediately following the H2b helix (Fig 1, S2 Fig). Also, only CNLs in Group B carry the previously described [44] putative myristoylation (Gly-2, Gly-3) and palmitoylation motifs (e.g., Cys-4, Ser-4, and others) in their N-termini. In contrast to the N-terminal domain of Mla10, Sr33, and Sr50, in which all four α helices appear to form CC structures, available algorithms [45] only predict formation of CC structures among N-termini of *At*-Col-0 CNLs for helices H1a and H1b.

The smallest group, Group A (comprising CC_R_–CNLs) include the three previously described activated disease resistance 1 (ADR1) homologs: ADR1, ADR1-L1, and ADR1-L2 [47,48]; two NRG1 homologs (AT5G66900 and AT5G66910) [24]; and a third NRG1-like protein annotated as DAR5 (AT5G66630) (Fig 1). Each of these six homologs contain RPW8-defined consensus sequences and four predicted α helices, yet these did not align with those of Groups B, C, and D (Fig 1, S1 Fig). In contrast to canonical CNLs, formation of CCs by N-termini of CC_R_–CNLs was predicted for three C-terminal α-helices but not the first α-helix.

### N-terminal fragments of Arabidopsis CNL receptors interact frequently but rarely self-associate

Heteromeric interaction between CC domains of the rice RGA4 and RGA5 CNLs and homomeric association of Mla10-CC have been previously detected using yeast 2 hybrid (Y2H) assays [22,26]. Therefore, we used Y2H to assess homo- and heteromeric interactions of the N-terminal CNL fragments. We cloned 56 DNA fragments encoding the N-terminal regions of CNLs, referred to hereafter as extended CC domains (ECCs) (Fig 1, S2 Fig, S2 File). Each ECC contains the entire N-terminus up to the predicted P-loop in the NB–ARC domain. This region includes the amino acids immediately preceding the predicted α-helices and the linker encompassing the pre-P–loop motif (Fig 1). Next, we tested their interactions in Y2H assays using initially two and later one pair of vectors, creating fusions to binding and activation domains (see Materials and methods).

Five ECC fragments showed homomeric associations (Fig 2B), while 123 heteromeric interactions were observed involving 39 ECCs. Interactions occurred at similar frequencies between sequence-related and sequence-unrelated ECCs. There seems to have been no evolutionary selection toward homomeric associations because homomerization was observed only for ECCs that also interacted frequently with other partners. If ECCs had evolved to facilitate CNL homodimerization (like previous studies have implied [22,26,32]), homomeric interactions should be prevalent, and heteromeric interactions between ECCs corresponding to close paralogs should be infrequent. However, 34 ECCs showed higher affinity toward at least one partner other than themselves. The large number of heteromeric interactions suggests that N-termini–mediated heteromerization is a common feature of CNLs.

**Fig 3:**
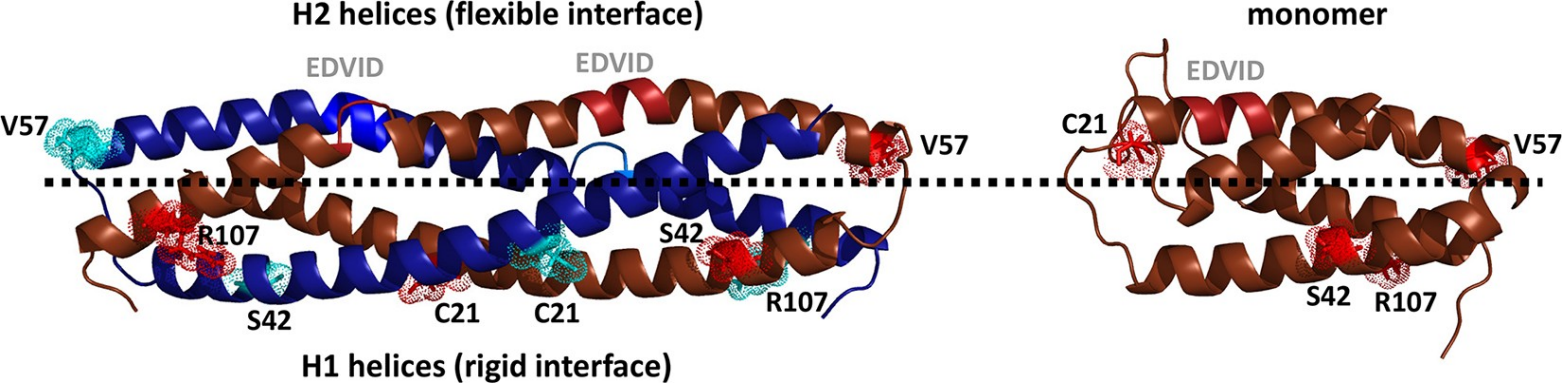
Ribbon representation of homodimeric (left) and monomeric (right) predicted structures for Group D CC domains built upon crystallographic data available for Mla10–CC and Sr33–CC. Four amino acids residues (represented with sticks and dots) conserved among frequently interacting homologs in Group D are located on the opposite side of the H2a–b turn. In the monomer, C21 resides in highly flexible region within the proximity of the EDVID motif. The thick dotted line delineates H1 helices (below) from H2 helices (above). The two different chains in the dimer (A and B) are colored in dark blue and brown. The monomeric structure is oriented to match the orientation of the dimer on the left. CC, coiled–coil; Mla10, powdery mildew resistance 10 in barley; Sr33, stem rust resistance 33 in wheat.

To better understand the molecular features within ECC domains that may be required or involved in the interaction, we correlated their sequence variation and the ability to interact in Y2H with their predicted CC monomeric and dimeric structures. First, all possible models of monomeric and dimeric structures that could be derived from available crystal structures were built. Based on knowledge and physical binding free energy calculations, the most probable models were retained (S1 File). The monomeric structural model (mono4α) resembles the four-helix bundle observed in Sr33–CC [32], whereas the dimer model resembles the intertwined CC structure (2α) observed in Mla10–CC [26]. Binding free energy calculations (S1 File) suggests that dimer configurations based on two mono4α domains have a higher binding free energy than the 2α monomers and are less likely. Second, we identified sequence variants that could be correlated with the ability to interact. For this, we focused on members of Group D, as these share overall high-sequence similarity but differ in their ability to interact (Fig 2B). As compared to rarely interacting ECCs in Group D, four amino acids were found to be conserved and unique to frequently interacting ECC domains of AT1G58390, AT1G58848, AT1G59218, AT1G58807, and AT1G59124: C21, S42, V57, and R107 (S1 Fig). To resolve their putative location on the protein surface, the positions of these residues were mapped on both the monomeric and dimeric 3D models (Fig 3). In the dimer, C21, S42, and R107 add up to six amino acids located on the H1 helices. All six residues are surface exposed and on the same face of the protein, while V57 has a more lateral location. In the monomer structure, C21 resides in a highly flexible region and may reach the proximity of the EDVID motif (Fig 3), whereas in the 2α dimer configuration, it resides in a rigid region, and the EDVID motif is no longer within reach. The genetic variation in Group D enabled correlating oligomerization potential to four amino acids residues that likely form a patch on the CC surface. However, the exact role of these residues in this process remains to be resolved in future studies and awaits elucidation of the protein structure.

### The ability to induce defense responses in Arabidopsis varies between CNL groups

Transient expression of a CC domain can induce necrosis and activate defense-related genes, thereby recapitulating induction of HR and defenses triggered by activated full-length CNLs [24,26,34]. To obtain a comprehensive assessment of abilities to induce cell death across CNLs, we transiently expressed all ECCs in *At*-Col-0 plants using the Tobacco Rattle Virus (TRV) system [49,50]. Arabidopsis is a good host for TRV supporting its systemic spread [51,52]; therefore, utilization of TRV allowed us to monitor not only necrosis but also disease resistance by assessing viral spread using reverse transcription PCR (RT-PCR) (Fig 2). Expression of ECCs in *At*-Col-0 plants induced various phenotypes. These responses varied from very mild leaf deformations to more severe deformations of the entire plant and, in extreme cases, necrosis or even death of infected plants (Fig 2C). In a separate category of response, full resistance was observed in which viral movement was blocked without any development of symptoms, and accordingly, TRV was not detected in leaves after inoculation. Different colors and numbers (zero through six; legend in Fig 2C) were assigned to these distinct categories for visualization and correlation analysis.

None of the members of Group D elicited a macroscopically visible response (Fig 2B). Expression of ECCs corresponding to the CN genes [17] belonging to Group E also did not induce any visible response in *At*-Col-0 plants. However, within Group C, two ECCs induced necrosis (AT3G46710 and AT3G46530), and one triggered mild plant deformations (AT3G14470) (Fig 2). In Group B, 14 of the 23 ECCs induced either necrosis or resistance in infected plants. Notably, all ECCs corresponding to CC_R_–CNLs (Group A) induced either necrosis or resistance. This Group includes AT5G66630, which our functional analysis classified as a third functional NRG1 homolog, raising the total number of CC_R_–CNLs in *At*-Col-0 to six. Besides CC_R_–CNLs, the ability to induce cell death was more prevalent (but not exclusive) for ECCs in Group B lacking the conserved EDVID motif (Fig 1). In conclusion, the ability of ECCs to trigger immunity varied over the different Groups from none (Groups D and E) to all members (Group A). Notably, whereas some ECCs triggered necrosis to various extents, others had the ability to fully block viral movement, showing that expression of an ECC alone can be sufficient to trigger disease resistance.

### The abilities of ECCs to oligomerize and induce cell death do not correlate

CC-mediated oligomerization may be involved in communication between sensor and actor CNLs at either activation or downstream-signaling stages. To assess the requirement of these interactions for immune/HR signaling, we investigated whether these properties were correlated within and between NLR Groups. Therefore, we mapped all known resistance specificities onto the ECC interactome in order to link known functions to the (in)ability of the respective ECCs to interact and/or induce necrosis/resistance. The number of interactions, including self-association, and the ability to induce necrosis or resistance between ECCs were not significantly correlated (r = −0.035; *p* ≤ 0.1). Apparently, the ability of ECCs to interact is independent of their ability to trigger immune responses. Accordingly, we found that many noninteracting ECC fragments triggered necrosis (e.g., ECC corresponding to AT1G12290, AT1G61180, and AT4G27190 or ECCs corresponding to CC_R_–CNLs: AT5G66900 or AT5G04720), while several ECCs showing extensive interactions lacked the ability to induce cell death (e.g., ECC corresponding to AT5G63020 or ECCs corresponding to the bottom clade within Group D; Fig 2C). Four out of five ECC fragments capable of homomerization triggered only mild or moderate plant responses. Notably, the same five fragments were also among the most frequent interactors with other partners, implying common features required for both associations and promiscuity in these interactions.

CC_R_–CNLs have been proposed to function as actors [24] for sensor CNLs to induce defenses following pathogen perception. This hypothesis corresponds with the ability of their N-termini to induce cell death [38,53]. In our screens, the frequency of interactions between CC_R_–ECCs (putative actors) and ECCs corresponding to canonical CNLs (putative sensors) were not different (*p* ≤ 0.05) from frequencies of interactions between ECCs of canonical CNLs. Because ECCs of several canonical CNLs induced cell death similarly to CC_R_–ECCs (implying that they also function as actors), we examined whether necrosis-inducing ECCs interacted more frequently than those that did not. Again, the frequencies of such interactions were not higher than expected from random distribution. Thus, despite the fact that in our assays, ECCs of several putative sensors, such as RPS5, ZAR1, or RPP13 homolog (note that the ECCs of RPS5 and ZAR1 did not induce cell death), interacted with several CC_R_–ECCs, we did not find evidence for higher prevalence of such interactions as compared to associations of ECCs corresponding to putative sensors. Moreover, the ECC of RPS2, a receptor whose function depends on three CC_R_–CNL homologs (ADRs) [38], did not interact with any of the ADR ECCs in our screens. Accordingly, based on the ECC interactome, we did not find evidence for preferential CC-mediated communication between putative sensors and actors; however, this does not preclude the possibility that sensors and actors may interact transiently in planta or via other domains, as shown for the RGA4/RGA5 pair in rice [22]. The extensive heteromeric interactions between ECC members from different classes is suggestive for such a signaling network.

### ECCs induce cell death in heterologous plant species

**Fig 4:**
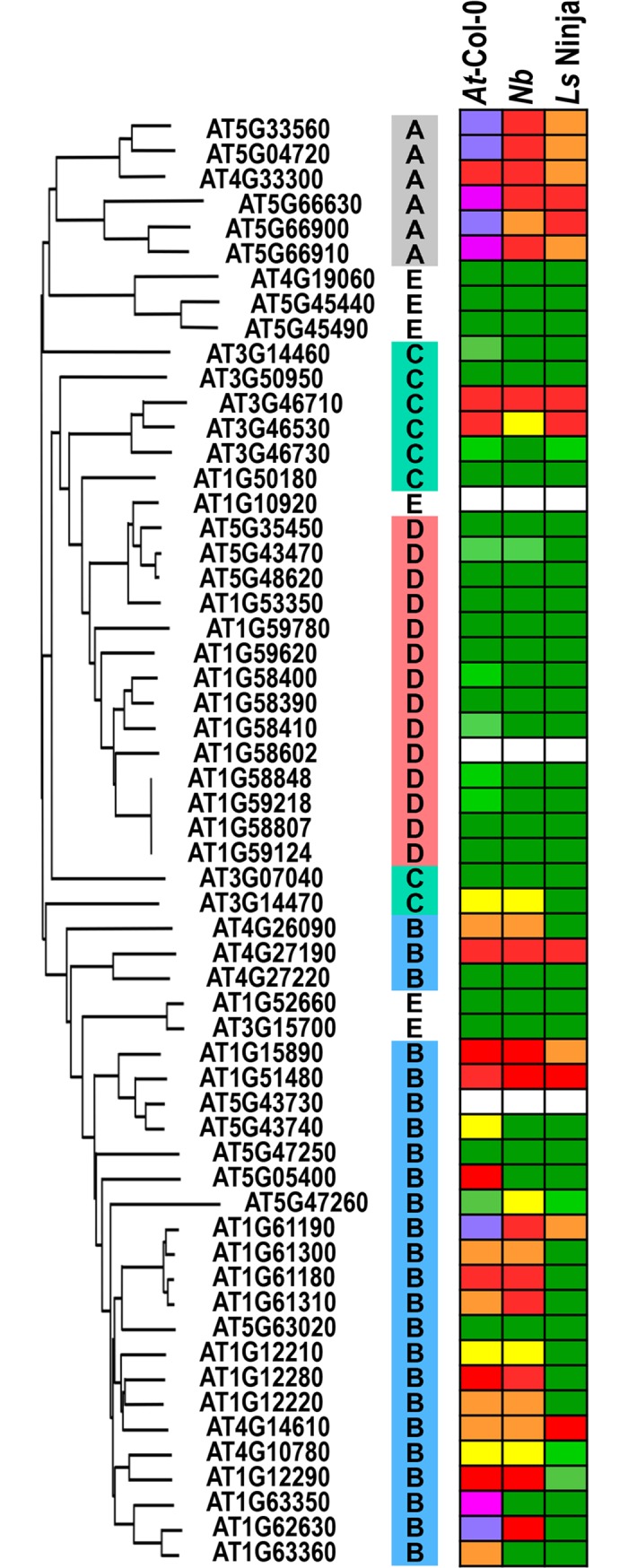
Responses to TRV-mediated transient expression of ECCs in *At*-Col-0, *Nb*, and lettuce cv. Ninja (*Ls* Ninja). All ECCs corresponding to CC_R_–CNLs (Group A) induce strong responses in the three species tested. Many sequence-related ECCs Group B members inducing a strong response in *At*-Col-0 also did so in *Nb* but rarely in lettuce. Persistence of the response across different species implies compatibility with downstream-signaling partners. Color coding for *At*-Col-0 represents phenotypes shown in Fig 2C: red, strong necrosis; orange, moderate necrosis/chlorosis; yellow, chlorosis; light green, mild chlorosis; green, no response. *At*-Col-0, *Arabidopsis thaliana* ecotype Columbia-0; cv., cultivar; ECC, extended CC domain; *Ls*, *L*. *sativa*; *Nb*, *N*. *benthamiana*; TRV, Tobacco Rattle Virus.

Genetic support for a CNL-signaling network comes from mutating hubs that compromise its activity. Despite mutating many putative interacting NLRs, no loss of necrosis was obtained upon ECC expression (Fig 2B, S3 File). This result is consistent with the resilience of a network in that other hubs can take over the function of the mutated node. To obtain evidence for a CNL-signaling network activated by *At*-Col-0 ECCs, we assessed the activity of these fragments in heterologous species. The rationale was that in a heterologous species, redundancy might be lower, as the node (representing a CNL) did not coevolve with the ECC that triggers the response. Occurrence of an ECC-induced response in evolutionarily distant species would imply conservation and compatibility of the immune-signaling network in these species. Furthermore, if a polymorphic response is obtained in a heterologous species, it might allow identification of the ECC-interacting partner(s). To evaluate the ability of CNLs to induce defenses in other species, we expressed all *At*-Col-0 ECCs in *Nicotiana benthamiana* (*Nb*) and in lettuce, *Lactuca sativa* (*Ls*) cultivar (cv.) Ninja (Fig 4).

ECCs corresponding to CC_R_–CNLs induced strong responses not only in *Nb*, as reported previously [24], but also in lettuce and the source species Arabidopsis. Out of the 16 Group B ECCs that triggered necrosis or resistance in *At*-Col-0, 13 induced necrosis in *Nb* and five did in lettuce, implying greater compatibility with downstream-signaling components in the source species and in *Nb* than in lettuce. This response is unlikely to be triggered by general toxicity because for some ECCs induction of necrosis was clone-specific and sometimes limited to one or two of the three species tested. The finding that many (and often the same) CC fragments triggered responses in distantly related species indicates conservation of compatibility with specific signaling partners.

**Fig 5:**
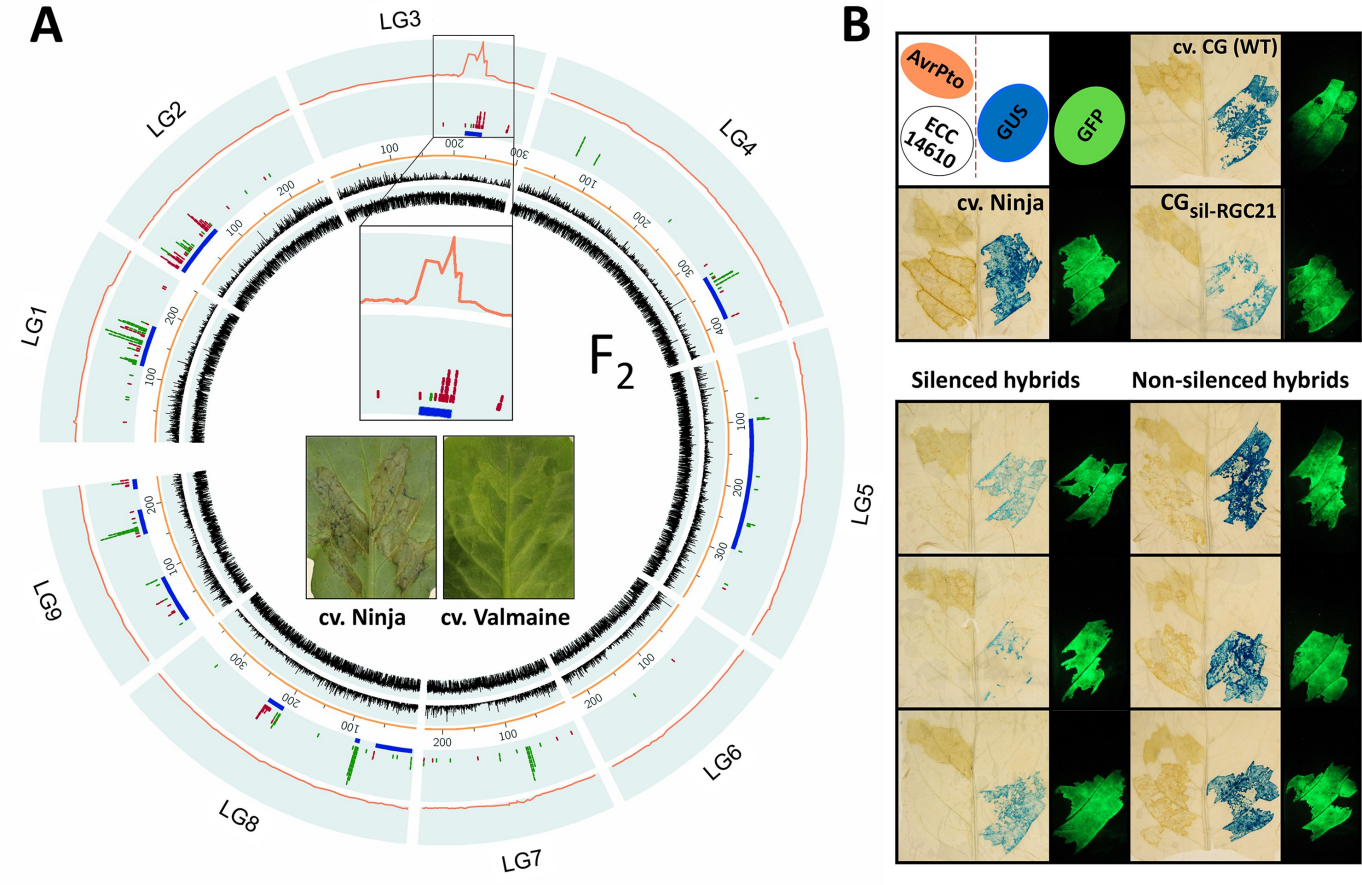
Member(s) of RGC21 CNL family in lettuce are required for necrosis induced by AT4G14610–ECC, as shown by genetic mapping and PTGS. (A) Nearly all of the variation in necrosis induction among the F_2_ plants derived from the cross between cv. Ninja and cv. Valmaine can be explained by a 22-cM interval (area enlarged inside the circle) in linkage group 3 (LG3) of the lettuce genome that contains multiple NLR-encoding sequences. Two black, innermost circles show density of repeats and genes in the lettuce genome, ruler distances are in cM, blue markings depict resistance-related genes, red markings CNLs, green makings TNLs, and the red outer line in the outer circle shows LOD scores from QTL analyses. (B) Silencing of *RGC21* family members compromised moderate necrosis induced by the ECC of AT4G14610 (ECC 14610) in cv. Ninja and CG_sil-RGC21_ hybrids. Decreased transient GUS expression (at comparable control GFP expression) among hybrids indicated silencing of *RGC21* because PTGS was triggered by ihpRNA containing both *RGC21* (Gene Bank accession number EU889315.1) and GUS sequences, as described previously [54,55][54,55]. Comparable intensities of necrosis induced by AvrPto in all plants tested showed that silencing of *RGC21* did not compromise necrosis elicited by other than the AT4G14610 ECC trigger. Expression of *GFP* and *GUS* reporter genes was captured three days post infection in fresh leaves and leaves cleared in 80% EtOH, respectively, using standard protocols [56,57]. CG_,_ Cobham Green; CNL, CC–NLR; cv., cultivar; ECC, extended CC domain; ihpRNA, interfering hairpin RNA; NLR, nucleotide-binding leucine-rich repeat receptor; PTGS, posttranscriptional gene silencing; RGC21, Resistance Gene Candidate 21; TNL, TIR–NLR.

Induction of cell death by ECCs in the three plant species allowed us to screen for an ECC/species combination that showed a polymorphic response upon *At*-Col-0 ECC expression. Interestingly, transient expression of ECC of AT4G14610 in lettuce cv. Ninja triggered clear cell death, whereas it did not elicit necrosis in cv. Valmaine (Fig 4, Fig 5A).

This differential response allowed genetic mapping to determine the genomic location of the potential AT1G14610-signaling partner in lettuce. In F_1_ hybrids between cv. Ninja and Valmaine and derived F_2_ plants, we observed intermediate phenotypes, indicating that either the ability to induce necrosis in cv. Ninja or the lack of a response in cv. Valmaine is due to incomplete dominance. Variation in plant response to the ECC of AT4G14610 expression was assessed in 75 individual F_2_ plants on a scale from 1 to 5. All plants were genotyped, and subsequent QTL mapping linked the variation to a single locus on linkage group 3 (LG3) of lettuce (Fig 5A). This locus contains candidate disease resistance genes, including sequences encoding TNLs and a large CNL family previously described as the Resistance Gene Candidate 21 family (*RGC21*) [55].

In a previous project, transgenic lettuce plants of cv. Cobham Green (CG), referred thereafter as CG_sil-RGC21_, were generated expressing an interfering hairpin RNA (ihpRNA) designated to trigger posttranscriptional gene silencing (PTGS) of *RGC21* family members [55]. The ECC of ATG14610 did not trigger necrosis in cv. CG; consequently, cell death assays could not be performed in CG_sil-RGC21_. Therefore, these silenced CG_sil-RGC21_ plants were outcrossed to cv. Ninja and to cv. Valmaine. Among 30 F_1_ hybrids derived from the cross between cv. Ninja and CG_RNAi-RGC21_, 14 were identified as silenced and 16 as not silenced for *RGC21*, which is as expected due to the hemizygous state of the transgene in CG_RNAi-RGC21_ (Fig 5B). All 16 nonsilenced hybrids showed a similar weak necrosis following expression of ATG14610–ECC as F_1_ cv. Ninja x cv. Valmaine F_1_ hybrids and many F_2_ plants derived from the same cross (Fig 5B). In contrast, similar to wild-type cv. CG plants, none of the 14 *RGC21*-silenced hybrids showed necrosis, indicating that one or more *RGC21* member(s) are required for induction of ATG14610-mediated cell death. Furthermore, none of the cv. Valmaine x CG_RNAi-RGC21_ hybrids showed necrosis despite many being identified as silenced. Notably, all accessions and hybrids described above responded with moderate necrosis to transient expression of bacterial effector AvrPto [58], indicating that the activity of *RGC21* member(s) in induction of cell death is specific to the ECC of ATG14610. The sequences and repertoire of *RGC21* paralogs in cv. Ninja are unknown, and the exact identity of the *RGC21* member(s) acting downstream of ATG14610 remains undetermined. However, reverse BLAST of *RGC21* sequences (Gene Bank accession number EU889315.1) to *At*-Col-0 sequences specifically identifies multiple members of Group C/D as closest homologs, indicating that ATG14610 requires member(s) of this Group for cell death induction in lettuce. From this experiment, we concluded that an *At*-Col-0 ECC requires CNL partner(s) to induce cell death and that despite the large diversity among NLR receptors, compatibility between CNLs can be retained across distantly related plant species.

### A highly variable fragment in the CC–NB linker is required for cell death induction

To identify the region/motifs within ECCs required for their activity, we searched for correlations between all 56 sequence variants and their ability to trigger cell death. Following the predicted four α helices, each ECC fragment contains a variable linker that separates the last α helix (H2b) from the pre-P–loop at the beginning of the NB–ARC domain. Alignments of all ECCs refined the consensus of a pre-P–loop motif among CNLs in Arabidopsis to V/IG x (8)L/I x (3)L and disclosed a cluster of charged amino acids within the linker region. This cluster, which we refer to as the “charged motif,” maps to positions −11 to −3 relative to the highly conserved VG residues of the pre-P–loop motif (S1 Fig).

**Fig 6:**
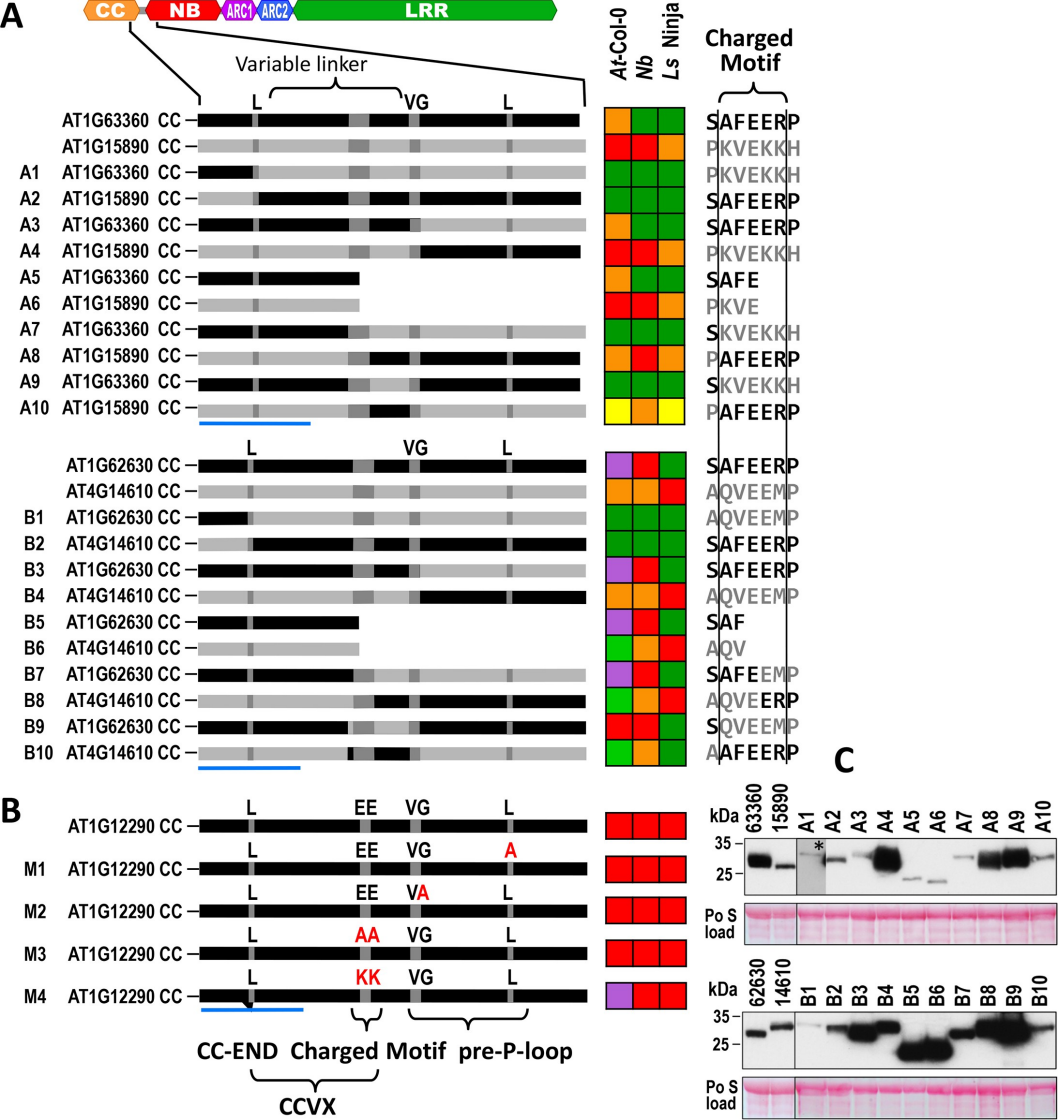
Deletion, swap, and point mutants used to identify the area within ECCs required for cell death induction and the response of *At*-Col-0, *Nb*, and lettuce cv. Ninja to their transient expression. (A) Two pairs of ECC clones, AT1G63360 and AT1G15890, shown in the top panel, and AT1G62630 and AT4G14610, shown in the bottom panel, were selected for targeted deletion and reciprocal swaps. The following residues (marked dark gray) were used as breaking points to produce swap mutants: last conserved heptad hydrophobic residue (L) of predicted CC domain CC-END, acidic (E or EE) residues within the area defined as “charged island,” and/or initial VG residues of predicted pre-P–loop. Charts to the right show the response of *At*-Col-0, *Nb*, and lettuce to transient expression of the respective ECCs. (The sequence of the variable linker corresponding to CCVX is shown in detail in Fig 7) (B) Point mutations introduced into ECC of AT1G12290. (C) Immunobloting of ECC proteins isolated from *Nb* leaves 48 h after agroinfiltration. Blots were probed with a primary rat anti-HA and a secondary goat anti-rat antibody fused to HRP. Po S load–loading control, * the signal corresponding to clone A1 was detected after a 5x extended exposure. Color coding for *At*-Col-0 represents phenotypes shown in Fig 2C, and color coding for reaction in *Nb* and lettuce was the same as described previously [58]: red, strong necrosis; orange, moderated necrosis/chlorosis; yellow, chlorosis; green, no response; horizontal blue line defines the most C-terminal α-helix of the CC domain. *At*-Col-0, *Arabidopsis thaliana* ecotype Columbia-0; CC, coiled–coil; CCVX, CC-variable amino acids residues; cv., cultivar; ECC, extended CC domain; HA, hemagglutinin; *Nb*, *N*. *benthamiana*; Po S, Ponceau S.

To determine exactly which regions in the ECC are required for *At* CNLs to trigger signaling, we generated a series of deletions, swaps, and point mutants involving cell death–inducing and non-cell death–inducing ECCs to delineate the region responsible for induction of cell death (Fig 6, S4 File). We focused on four ECCs in Group B because their high homology and accurate alignment allowed precise swaps, yet they had different phenotypes to differentiate the output. The following amino acids residues were used as break points to create chimaeras: the last conserved hydrophobic residue of heptad repeat of H2b (referred thereafter to as CC-END), the start (VG residues) of the pre-P–loop, and the charged motif (Fig 6). Because the selected ECCs induced necrosis in more than one plant species (Fig 4), we examined the plant response in *At*-Col-0, *Nb*, and in lettuce cv. Ninja. Four wild-type clones and all chimeras were fused to C-terminal hemagglutinin (HA) tag to evaluate their expression in *Nb* (Fig 6A).

Reciprocal swaps at CC-END between ECCs (corresponding to two pairs of CNLs: AT1G63360 and AT1G15890, and AT1G62630 and AT4G14610) surprisingly resulted in chimaeras that lost their ability to induce cell death in any species (A1 and A2, B1 and B2; Fig 6A). Accordingly, clones that previously did not induce necrosis (like ECC of AT1G63360 in *Nb* and in lettuce or ECC of AT1G62630 in lettuce) did not gain this ability after introducing the CC-END-linker-pre-P–loop fragment from a necrosis-inducing clone (A1 and B1, respectively; Fig 6). This indicated that regions upstream and downstream of CC-END were required but alone were insufficient for induction of cell death. Furthermore, these results showed that compatibility between both regions was essential for induction of cell death.

A reciprocal swap at the VG residues (pre-P–loop) between the same two pairs of ECCs (A3, A4, B3, and B4; Fig 6A) did not affect their ability to induce cell death nor their patterns across the three plant species, implying that the pre-P–loop itself is not required for elicitation of cell death. Deletions in ECCs at the charged motif did also not affect the ability or patterns of necrosis induction in three clones (clones A5, A6, and B5) but eliminated growth deformations triggered by the ECC of AT1G14610 in *At*-Col-0 (clone B6; Fig 6A). This implied requirement of the fragment between CC-END and the charged motif for cell death induction. The charged motif itself was not essential for cell death induction but clearly modulated the response. Indeed, substitutions G158A at the beginning of pre-P–loop and E/E148/149A/A within the charged motif in the ECC of AT1G12290 did not compromise cell death induction (M1–M3; Fig 6B) in *At*-Col-0 plants, but substituting the same EE residues with positively charged KK potentiated the immune response and made *At*-Col-0 fully resistant to viral infection (clone M4; Fig 6B).

Reciprocal swaps at the charged motif between ECCs corresponding to AT1G63360 and to AT1G15690 (the exact position of the swaps is shown in the left panel in Fig 6A) resulted in the elimination of plant deformations induced by the former in *At*-Col-0 and weakened the response induced in all three plant species by the latter (A7 through A10; Fig 6). A similar swap between ECCs corresponding to AT1G62630 and AT4G14610 eliminated the response induced by the latter in *At*-Col-0 (B8 and B10, Fig 6A). Swapping the entire charged motif between the ECC of AT4G14610 with the ECC of AT1G62630 compromised cell death induced in lettuce and *At*-Col-0 but not in *Nb* (B10, Fig 6A). This implied that the charged motif, besides modulating the strength of the response, may also determine host specificity.

To exclude the possibility that a lack of responses was due to a lack of stability of the chimaeras, protein accumulation of wild-type ECCs and their derived variants was assessed in *Nb* leaves using immunoblotting (Fig 6C). Full-length ECCs migrated at an apparent size of 25 to 35 kDa, which is slightly larger than the predicted sizes of 22–23 kDa (including HA tag) and might be attributed to the high content of hydrophobic residues (V, I, and L). Protein accumulation levels varied between constructs and appeared to be slightly reduced for four clones swapped at CC-END (A1, A2, B1, and B2). Notably, A3 or A10 triggered plant responses despite comparably low accumulation levels; this implied that the amount of protein in the aforementioned swaps should have sufficed to trigger a response in the three plant species tested. Interestingly, ECC At1G63360 and the A4, A8, and A9 proteins ran at an apparent increased molecular weight, suggesting an unknown posttranslational modification of these fragments. The reduced accumulation of A1, A2, and A5 through A7, A10, B1, and B2, as compared to the WT protein, may indicate a stabilizing role for this region and necessity for compatibility between this motif and the N-terminal portion of the fragment. To conclude, the lack of responses following expression of the chimaeras was likely due to a loss of activity rather than a reduced accumulation or stability of the produced protein.

**Fig 7:**
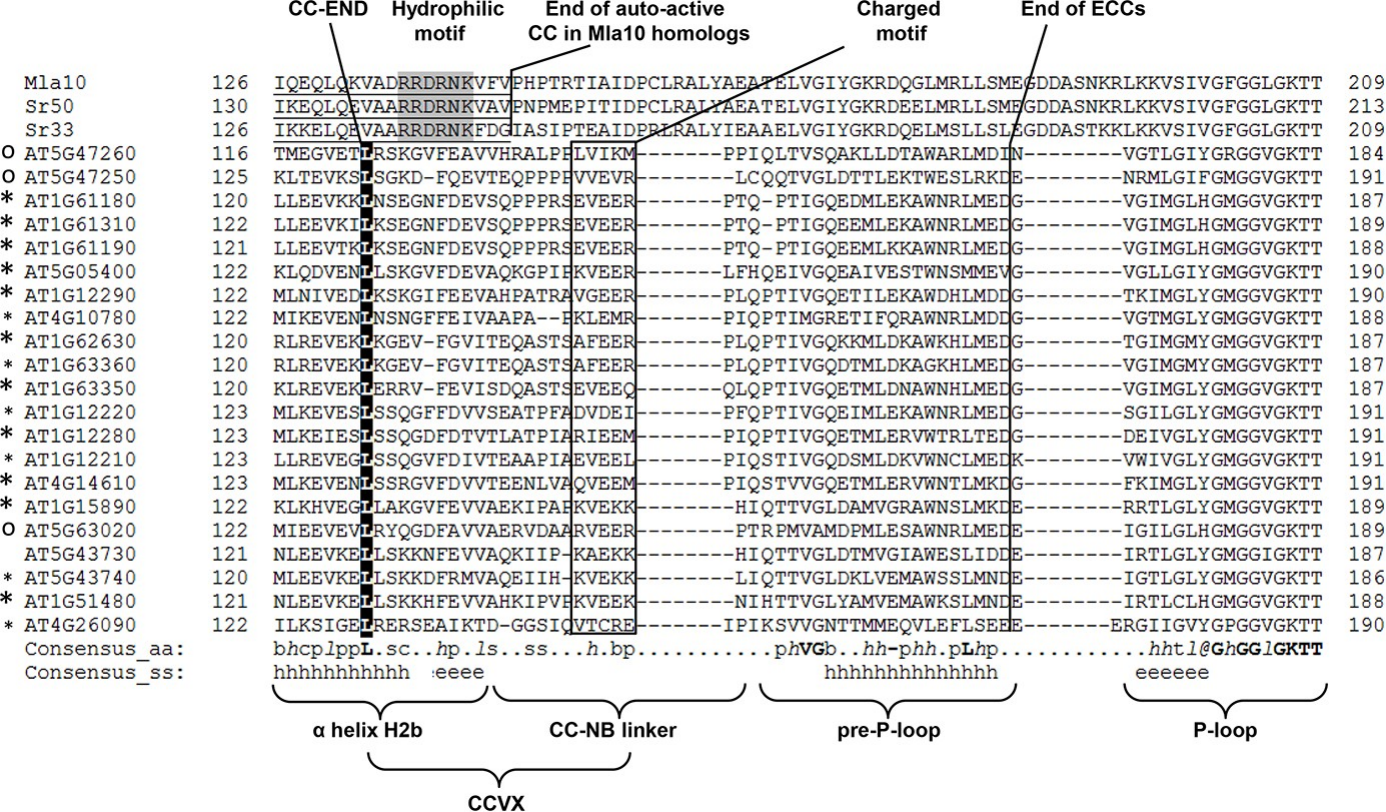
Alignment of the area following α-helix H2b in three Mla10 homologs and a representative subset of *At* CNLs. The alignment shows the differences in the position of the hydrophilic motif previously identified by Cesari and colleagues [34] and the charged motif identified in this study. The hydrophilic motif among homologous monocot CNLs is located within the predicted helix H2b (underlined), whereas charged motif is outside of predicted α-helix within the predicted CC–NB linker. The fragment between CC-END and charged motif, CCVX, is required for induction of cell death, but its sequence is variable across different CNLs in *At*-Col-0. ECCs capable of inducing cell death or deformation in *At*-Col-0 plants are marked with larger and smaller asterisks; “o” indicates no reaction. Alignment was created using PROMALS3D [46]. *At*-Col-0, *Arabidopsis thaliana* ecotype Columbia-0; CC, coiled–coil; CCVX, CC-variable amino acids residues; CNL, CC–NLR; NB, nucleotide-binding.

From these experiments, we concluded that the integrity of a variable stretch of 16 to 18 amino acids (referred hereafter to as CC-variable amino acids residues [CCVX]) following the last hydrophobic residue in helix H2b is required (but insufficient) for induction of cell death (Fig 6 and Fig 7). The potency of the CCVX fragment in induction of cell death can be modulated by its charge, implying the involvement in electrostatic intra- or intermolecular interactions. Because of its involvement in cell death elicitation, the charged motif may be analogous to the hydrophilic motif identified in three homologous CNLs in monocots [34] despite being localized in a slightly different position relative to H2b (Fig 7).

## Discussion and conclusions

Among STAND receptors, the presence of an N-terminal CC domain is exclusive to CNL pathogen receptors in plants [2,37]. Our interactome analyses revealed a high tendency of ECCs to form heteromeric interactions as compared to homomerization, suggesting functional importance of associations involving different CNLs. Approximately two-thirds of the ECCs associated with more than one ECC, which implies functional redundancy among the CNL interactome. Consistently, knockouts of multiple NLR interacting partners in Arabidopsis did not compromise the ability of a specific *At*-Col-0 ECC to trigger an immune response (S3 File). In lettuce, however, a single dominant CNL locus (*RGC21* locus) could be associated with the ability of AT4G14610–ECC to trigger HR. Existence of a CNL network in Arabidopsis is consistent with the availability of the many putative sensor- and actor-type receptors [39]. In tomato, an NLR network is defined [39] in which sensors signal via downstream actors. Whether the tomato sensor NLRs form similar heteromeric interactions as the *At*-Col-0 ECCs (Fig 2A) needs to be resolved. Unlike in tomato, involving CC_R_–CNLs as actors (or helpers) [39], the partnering receptor of AT4G14610–ECC in lettuce appeared to be an EDVID-type CNL (Fig 2B). Nevertheless, the ability of NLRs to transduce their signal via various partners seems a recurring theme in plant immune signaling because not only NLRs involved in ETI form networks but also *At*-Col-0 LRR receptor kinases involved in MTI show an extensive network of interactions and functional dependencies [59]. A network provides resilience to perturbation and manipulation by pathogens and facilitates compatibility to adjust to new recognition specificities [40].

Identifying a lettuce CNL as a downstream-signaling partner for AT4G14610–ECC also has implications for CNL evolution. Canonical NLRs evolve rapidly, being highly diverse across different taxa, yet despite approximately 100 million years following the separation of superrosid (Arabidopsis) and superasterid (lettuce) lineages [60], the identified NLR pair retained a functional relationship. Interactions between Group B and Group C/D members were frequent (Fig 2B). Because compatibility between the AT4G14610–ECC and RGC21 CNL were retained, specific molecular features must be conserved within the N-terminal fragment. For Mla10 homologs, both the predicted α-helices and the hydrophilic motif was proposed to be involved in CC–CC interactions [26,32]; hence, the evolutionarily conserved feature retaining compatibility between ECCs may simply be the ability to oligomerize.

Adaptation of the TRV-based expression system enabled functional analysis of the ECCs in the source plant, Arabidopsis, in *N*. *benthamiana*, and in lettuce. Induction of cell death by ECCs was not unique to CC_R_–CNLs (the number of which we increased to six by the identification of AT5G66630) but could also be triggered by approximately half of the ECCs present in the repertoire of CNLs in *At*-Col-0. Notably, five ECC fragments were able to trigger full immunity in *At*-Col-0 to TRV infection. It is unclear whether these fragments themselves function as actors or represent sensors signaling via an actor. Overexpression of some confirmed sensor ECCs, such as the Group B members RPS2 and RPS5, triggered a mild reaction but no resistance. Overexpression of ECC domains from putative sensors from Group C (RPM1 and ZAR1) did not trigger any response like most other members of this Group and from Group D. Together, these findings show that an unambiguous distinction between actors and sensors based on the ability of their ECC to trigger immune responses may not be possible. Nevertheless, these observations together demonstrate that the ECC represent the “effector” domain of a CNL that is sufficient to trigger full resistance, supporting recent conclusions of Jacobs and coworkers [27]. Furthermore, our findings are in agreement with loosening the association between the CC domain and the remainder of the CNL following conformational rearrangements upon activation [15]. Such a mechanism is analogous to release of the CARD of APAF1 or metazoan NLR receptors following their activation [5,10,61](S3 Fig). However, in contrast to the CARD in metazoan receptors, the CC is capable to signal and interact autonomously and apparently does not require an NB–ARC to trigger defense responses when heterologously expressed in planta.

A recent study revealed an increased binding affinity of the CC domain of RPM1 toward CC–NB–ARC domain variants that harbored autoactivating mutations [36]. Notably, this increased affinity was only observed using extended CC domains containing the NB–ARC linker encompassing the charged motif identified in our study. This finding implies that ECCs may transactivate full-length downstream CNLs in at least two distinct ways. First, (as indicated by our Y2H data), the CC of one receptor may interact with the CC of the downstream partner, thereby relieving the negative regulation of its NB, allowing it to adopt an activated ATP-bound conformation. Second, the ECC linker may directly interact with the NB domain of a partner CNL, thereby activating the receptor. In either scenario, a cascade of transactivation may be initiated because the released ECCs could potentially activate additional CNL receptors. The presence of two interaction surfaces (CC–CC and CC–NB) would stabilize the complex formed, facilitating the formation of multimeric (yet self-terminating) complexes similar to heteromeric inflammasomes described for mammalian NLR family apoptosis inhibitory protein 2 (NAIP2) and NLRC4 [7,10] (S4 Fig). In contrast to the metazoan NLRs in which the NB triggers transactivation and multimerization, CNLs appear to be able to also employ the CC domain to transactivate other CNLs and possibly nucleate NLR multimerization (S4 Fig). After activation and reaching initial proximity, further association of monomers could involve the NB–ARC domain of the full-length receptor. Such a mode of association is consistent with oligomerization of other STAND receptors, such as APAF1, in which the interface between monomeric subunits is complex and involves different parts of the receptor [6].

We demonstrated that variable area (CCVX) (Fig 6, S2 Fig) located in CC–NB linker is involved but not sufficient for induction of cell death. Chimeras swapped at the last hydrophobic residue of the CC lose their ability to induce necrosis, implying that compatibility between the C-terminal part of the H2b helix and the CCVX with the remainder of the CC is required to induce cell death. This requirement implies that certain structural features are needed to trigger cell death, which is consistent with the hypothesis that the CC may share its origin with metazoan death folds [37]. Accordingly, the hydrophilic motif [34] and the CCVX may be structural motifs required for CC folding and function.

The observation that substitution of EE residues within the charged motif to positively charged lysines enhanced the plant immune response opens up an interesting possibility to engineer CNL receptors to enhance their performance after activation in a more subtle manner than mutations in the NB–ARC domain that typically trigger autoactivity or loss of function [62,63]. Possibly, a potentiated ECC might confer a stronger defense against pathogens. The transfer of resistance genes (encoding NLRs) from one species to another is believed to require compatibility of upstream (such as decoys) and downstream-signaling partners [64]. If endogenous CNLs are immediate signaling partners of receptors, then the observed interspecies compatibility is surprisingly high, as suggested by the similar patterns of HR induction in unrelated plant species (Fig 4). Notably, CCs of Mla10 homologs from a monocot induce cell death in the dicot *Nb* [26,32,34]. Therefore, it is surprising that their structurally closest homologs in *At*-Col-0 (Groups C and D) (Fig 2B, S1 Fig) rarely induced necrosis. Whether this lack of functional homology can be attributed to convergent evolution or represents diversification in the ability of the ECCs to trigger defenses among domains of a common origin remains a question for future study. It will also be interesting to test whether compatibility of ECC signaling in heterologous species solely involves compatibility to the corresponding CNL, or whether there are other requirements. If not, this would suggest that CC-mediated oligomerization is the main factor in CNL immune signaling, as proposed in our model in S4 Fig.

ECC-driven formation of multimeric CNL complexes (similar to apoptosomes or inflammosomes in metazoa) [65] results in the formation of the active resistasome (or NLRsome), thereby providing a platform for communication between sensor and actor NLRs. The ability of ECCs to recruit other receptors and the self-terminating nature of a circular complex may enable precise control of defense induction, which is consistent with the quantitative nature of plant immune responses.

## Materials and methods

### Sequence bioinformatics and molecular modeling

Protein sequences were profiled for their predicted physicochemical profiles, as previously described [66–68]. Profiles were raised for linker, coil–coil, intrinsic disorder, secondary structure, contact, and turn-forming propensities [69–73]. For each profile, several methods were used, and the consensus was built to increase the prediction reliability, as described in the references above. The alignment was refined using Mla10–CC, Sr33, and Rx–CC structural data [26,32,33]. Sequence-clustering/phylogenetic tree–building was carried out using structural words weighting and variability analysis. Remote homology 3D models of CC domain structures were built, as described [26,74]. In essence, the alignments of target sequences were optimized, incorporating predicted secondary structure profile data as well as other predicted physicochemical profiles, followed by threading with SLIDE [75]. Molecular modeling was performed using Discovery Studio software (Accelrys-Dassault Systèmes) and Modeler v9.18 [76]. Along the conserved regions of the proteins, coordinates were assigned using standard Modeler coordinate transfer functions, while insertion loops were generated randomly and chosen by energy- and steric-based procedures. Generated loops were brought to local minima using a divide and conquer strategy, including recursive rounds of energy minimization and/or simulated annealing. The global model generated was further subjected to energy minimization, followed by global and local quality check using MetaMQAP [77], MolProbity [78], and PROCHECK V.3.4.4 [79] for crystallographic standards compliance. The overall structural optimization was performed with NAMD [80]. Molecular dynamics simulation experiments were then performed with Amber16 [81] using ff14SB force field at 300 K and 1 bar. The standard protonation state at physiological pH (7.4) was assigned to the ionizable residues using H++ server [82]. Dimer structures were solvated with TIP3P waters in an octahedral box. Periodic boundary conditions and Ewald sums (grid spacing of 1 Å) were used to treat long-range electrostatic interactions. The nonbonded cut-off distance was maintained at 12 Å, and the temperature and pressure were controlled by Langevin thermostat and Berendsen barostat with coupling constant of 1 ps. The quality of the simulations was assessed by analyzing the potential energy, root-mean-square deviations, and root-mean-square fluctuations profiles from molecular dynamics simulations. Binding free energies were calculated using molecular mechanics-based MM/PB(GB)SA methods, as implemented in Amber16 package specialized scripts [81,83] and knowledge-based Prodigy method [84]. Visual inspection and protein structure graphics were performed with PyMol (PyMOL Molecular Graphics System, v1.8 Schrödinger, LLC) and VMD [85]. Hydrophobicity profiles (Fig 1) were calculated using the Protscale server (web.expasy.org) using hydropathicity scale [86] and sliding window size of three residues.

#### Cloning of ECCs, Y2H assays, and data analysis

Sequences encoding CNLs were selected using published data [17] and online resources (TAIR; http://www.arabidopsis.org). ECC-encoding DNA fragments were amplified from *At*-Col-0 cDNA using primers containing a universal 5’extension (Frame B) to enable Gateway cloning into pDONR207 vector (Cat. No. 11791–020; ThermoFisher Scientific, https://www.thermofisher.com/). pDONR207 entry clones were transferred to Gateway-compatible destination vectors for Y2H analysis. Two systems were used to evaluate the interactions: (1) a standard Matchmaker GAL4 Yeast System (Cat. No. 11791–020; Invitrogen; http://www.invitrogen.com) utilizing pLAW10 and pLAW11 vectors in which the GAL4-binding and -activation domains were fused to the N-termini of the ECCs and (2) pGBKC and pGADC vectors modified to enable compatibility with Gateway Frame B [87], in which the GAL4-binding and -activation domains were fused to the C-termini of ECCs. Of the 48 fragments initially screened using pGBKC and pGADC vectors, only one-third of the interactions (and no new ones) that were observed with the Matchmaker GAL4 Y2H were detected. Therefore, the Matchmaker GAL4 system was used in all subsequent analyses. Interactions were assessed for their ability to grow on selective media lacking His and adenine and mapped using Cytoscape [88]. Matings for each prey versus bait combination were repeated four times.

### Expression of ECCs and plant analysis

ECCs were expressed in planta plants using the TRV system [89], in which pTRV2-attR2-attR1 was modified to enable expression of recombinant sequences by adding a coat protein promoter from Pea Early-Browning Virus (PEBV) [50,90]. The PEBV CP promoter [91] was amplified using primers 5′ATATGGTTACCGCACACAAGGTTAAAAACGCTG and 5′ATCTCGAGTTAGCTAGTTAGGCCTCTCGTTAACTCGGGTAAGTGA (restriction sites are underlined) and after digestion with *Bst*EII and *Xho*I ligated to the pTRV2-attR2-attR1 vector digested with the same enzymes. Subsequently, the vector was converted into a Gateway compatible vector by introduction of a *ccdB* Frame B cassette (Gateway Conversion System; Cat. No. 11828029, ThermoFisher Scientific, https://www.thermofisher.com/) into the *Stu*I site. To facilitate protein detection, a derived vector was created that contained the sequences encoding a human influenza HA tag introduced between *Stu*I and *Xho*I sites. The vector contains a PEBV CP promoter, followed by a Gateway conversion site, as described above. Clones containing swap- and substitution-mutations were custom synthesized as gBlocks (IDT; https://www.idtdna.com/) flanked by *attB1* and *attB2* recombination sites, and cloned to pENTR207 vector via a BP reaction. All ECCs and their mutants were recombined into this modified pTRV2-attR2-attR1 vector and transferred to *Agrobacterium tumefaciens* C58 (Rif^R^) [92]. Inoculation assays were performed as described for *At*-Col-0 [93], *Nb*, and lettuce [94]. Prior to infiltrations, suspensions of *A*. *tumefaciens* (OD_600_ = 0.5) harboring plasmids encoding TRV RNA1 [89] or TRV RNA2 were mixed in a 1:1 ratio. Col-0 plants were evaluated 4, 6, and 8 days post inoculation (dpi), whereas the reactions in *Nb* and lettuce were scored 2 and 3 dpi. Phenotypes reported in Fig 1C, Fig 4, and Fig 6 reflect the final score at the last day of observation. The assays in Col-0 were replicated at least twice for each clone using at least three plants in each replicate, the assays in *Nb* and lettuce were replicated at least twice and involved at least two leaves, each inoculated in two places. The presence of TRV, indicative of systemic infection, in *At*-Col-0 plants was detected by standard RT-PCR using primers design to amplify a fragment of the *At*-Col-0 actin-2 gene (control; AT3G18780) and the sequences encoding the coat protein of TRV: TAACCCAAAGGCCAACAGAG and GGGCATCTGAATCTCTCAGC for actin-2 and ACGATTCTTGGGTGGAATCA and CGGTGCAGATGAACTAGCAG for TRV CP (AF406991). Total RNA was extracted from *At*-Col-0 leaves using RNeasy Plant Mini Kit (Cat. No 74904, Qiagen; https://www.qiagen.com/), and cDNA used for PCR was synthesized using SuperScript II Reverse Transcriptase (Cat. No 18064014) and random hexamers. Detection of HA-labelled proteins was performed as described [95]. Standard statistical tests involving Pearson Correlation and Chi-Square tests were applied to data analysis.

### Genetic mapping in lettuce

For segregation analysis, a population of 75 F_2_ individuals derived from the cross between cvs. Ninja and Valmaine was tested for the response to the ECC of AT4G14610. This population was genotyped using next-generation sequencing (GBS) [96]. In brief, DNA was extracted from each individual, digested with *Ava*II to reduce the genomic complexity, and ligated to unique barcoded adapters (Truco and colleagues, in preparation). All samples were pooled and sequenced using Illumina HiSeq 4000. After sequencing, TASSEL [97] was used for demultiplexing, read mapping against the lettuce reference assembly, and SNP calling. Custom scripts (https://github.com/alex-kozik/atgc-xyz.) were used to obtain single haplotypes per scaffold. Scaffold-based haplotypes were used to construct a genetic map using MSTmap [98]. QTL analysis was conducted using WinQTL Cartographer and Composite Interval Mapping [99]. Significance threshold at *p* ≤ 0.05 was calculated by permutation analysis (1,000 permutations). The graph in Fig 7A was created using CIRCOS (http://circos.ca).

### Silencing of the *RGC21* CNL family in lettuce

Cv. CG was transformed with transgene LserNBS02_NB_RNAi (chr 3) to producing ihpRNA corresponding to fragments of a *RGC21* family member and the *uidA* gene [55]. Seedlings derived from two independent transgenic plants were tested for silencing by assessing a decreased transient GUS expression, as described previously [54]. Six individuals exhibiting silencing were crossed to cvs. Ninja and Valmaine. Hybrids were identified based on distinct morphology as compared to cv. Ninja used as a maternal parent and repeatedly tested for silencing using transient GUS expression. Progenies of two transgenic CG plants showed 1:1 segregation for the silencing phenotype and were tested for the response to ECC of AT4G14610, as shown in Fig 7B.

## Supporting information

S1 Fig Structure-based alignment of the N-terminal fragments of CNLs and CN proteins in *At*-Col-0. Residues common to frequent interactors (Fig 2B) are highlighted in dark blue. The first letter preceding the name of each gene indicates its corresponding Group. Hydrophobic amino acids within CC domains are highlighted in yellow, and A and D heptad positions are displayed in bold. *At*-Col-0, *Arabidopsis thaliana* ecotype Columbia-0; CC, coiled–coil; CN, CC–NB; CNL, CC–NLR.
(PDF)

S2 Fig Alignment of the N-terminal domains of the five Groups of CNLs defined by their predicted structural features. Amino acid residues in red font form predicted αhelices; residues in blue compose predicted beta sheets. CNL, CC–NLR.
(PDF)

S3 Fig **Neighbor-joining phylogenetic trees containing bootstrap values produced using either CC–NB–ARC (A) or the NB–ARC (B) sequences of CNLs in *At*-Col-0.** The color-coded Groups identified by structure globally overlap with Groups identified previously (delineated with braces on the right of each three) based on alignment of NB–ARC domains, analysis of conserved motifs, and intron/exon distribution [17]. The dotted red lines depict clones assigned differently between the two alignments. Colors present different Groups identified by structure-based alignment of the CC domain: gray, Group A (CC_R_); blue, Group B; turquoise, Group C; peach, Group D; white, Group E. Color coding is the same as in Figs 2 and 4 and S2 Fig. The tree was produced using MEGA [100]. *At*-Col-0, *Arabidopsis thaliana* ecotype Columbia-0; CC, coiled–coil; CNL, CC–NLR.
(PDF)

S4 Fig Activation and formation of active NB-signaling complexes by metazoan APAF1 and NOD receptors, the latter represented by NAIP2 and NLRC4 and by CNLs in plants. Perception of an initial signal triggers structural rearrangement to expose the NB–ARC domain in APAF1 and NOD receptors or the CC in sensor type CNLs in plants. Left: in case of APAF1, present as single isoform in the cell, the NB–ARC domain of an activated APAF1 monomer mediates oligomerization involving NB–ARC domains of additional APAF1 molecules [65]. NB–ARC–mediated oligomerization leads to the formation of a heptameric apoptosome and enables association of N-terminal CARDs and their interaction with protocaspase 9 to trigger pcd. Middle: upon activation of NAIP2, its exposed NB–ARC domain transactivates and associates with NOD receptor NLRC4 [10]. The hence exposed NB–ARC of NLRC4 triggers sequential transactivation of and associations with nine other NLRC4 monomers to produce an inflammasome composed of one NAIP2 and 10 NLRC4 subunits. BIR domains of NAIP2 and CARDs of NLRC4 interact and associate with caspases similar to CARDs in the APAF1 apoptosome. Upon activation, CC domains of CNLs in plants become exposed and trigger activation of other CNLs. Initial interactions between the monomers are likely mediated by CC domains, but similar to other STAND receptors, formation of signaling-competent complex requires interaction between the NB–ARC domains. Because the number of different CNLs may exceed several hundred in a single plant cell, other CNL receptors may also be activated and recruited to form heteromeric CNL NLRsomes. APAF1, Apoptotic Protease Activating Factor 1; BIR, Baculovirus inhibitor of apoptosis protein repeat; CARD, caspase-recruitment domain; CC, coiled–coil; CNL, CC–NLR; NAIP2, NLR family apoptosis inhibitory protein 2; NLR, nucleotide-binding leucine-rich repeat receptor; NOD, nucleotide-binding oligomerization domain; pcd, programmed cell death; STAND, signal transduction ATPases.
(PDF)

S1 File Molecular modeling, binding free energy estimations, and dimer stability analysis for Group D CC domains. CC, coiled–coil.
(PDF)

S2 File Amino acid sequences of the N-terminal fragments of CNL receptors in *At*-Col-0. The fragment highlighted in gray corresponds to the ECC used in functional analyses. The P-loop is marked in red. *At*-Col-0, *Arabidopsis thaliana* ecotype Columbia-0; CNL, CC–NLR; ECC, extended CC domain.
(PDF)

S3 File List of Arabidopsis KO mutants analyzed for an altered HR output upon expression of ECCs or an Avr that triggers immune signaling. Avr, avirulence factor; ECC, extended CC domain; HR, hypersensitive response; KO, knockout.
(PDF)

S4 File Sequences of swap, deletion, and point mutants in ECCs used to identify the area involved in cell death induction. ECC, extended CC domain.
(PDF)

## Abbreviations

ADR1: activated disease resistance 1
APAF1: Apoptotic Protease Activating Factor 1
*At*-Col-0: *Arabidopsis thaliana* ecotype Columbia-0
CARD: caspase-recruitment domain
CC: coiled–coil
CCVX: CC-variable amino acids residues
CN: CC–NB
CNL: CC–NLR
ECC: extended CC domain
dpi: days post inoculation
ETI: effector-triggered immunity
LRR: leucine-rich repeat
HR: hypersensitive response
ihpRNA: interfering hairpin RNA
MAMP: microbe-associated molecular pattern
Mla10: mildew resistance 10
MTI: MAMP-triggered immunity
NAIP2: NLR family apoptosis inhibitory protein 2
NB: nucleotide-binding
NLR: nucleotide-binding leucine-rich repeat receptor
NOD: nucleotide-binding oligomerization domain
pcd: programmed cell death
PEBV: Pea Early-Browning Virus
PTGS: posttranscriptional gene silencing
RGC21: Resistance Gene Candidate 21
RLK: receptor-like kinases
Rx: virus X resistance
Sr33: stem rust resistance 33
TIR: Toll/interleukin-1 receptor
TNL: TIR–NLR
TRV: Tobacco Rattle Virus
Y2H: yeast 2 hybrid
