# Supplementary material for: Genome-wide functional analyses of plant coiled–coil NLR-type pathogen receptors reveal essential roles of their N-terminal domain in oligomerization, networking, and immunity
Source: PLoS Biol. 2018 Dec 12;16(12):e2005821. doi: 10.1371/journal.pbio.2005821 (PMC6312357; doi:10.1371/journal.pbio.2005821)
Supplement: S2 Fig — Amino acid residues in red font form predicted αhelices; residues in blue compose predicted beta sheets. CNL, CC–NLR. (PDF) [file pbio.2005821.s002.pdf]

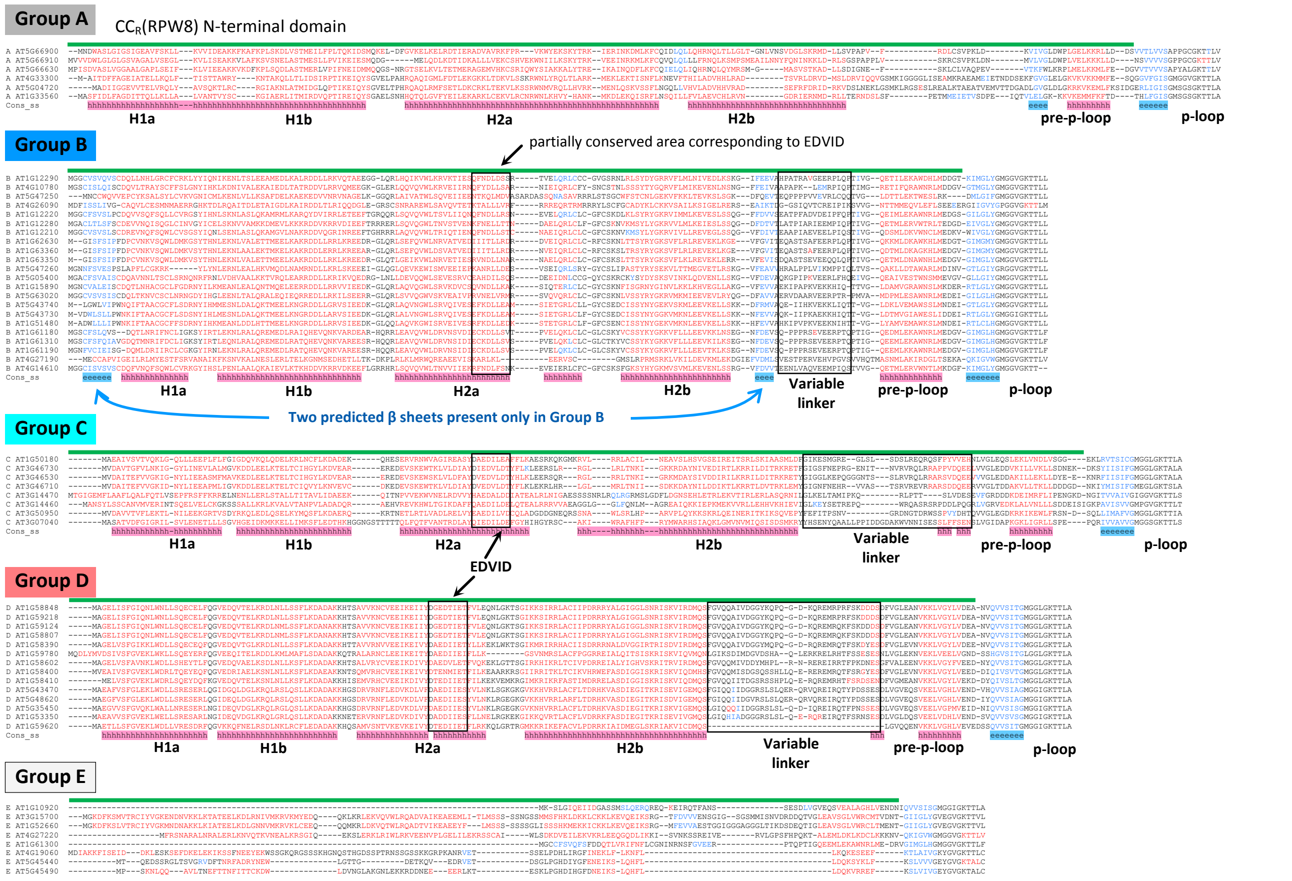

Group A
CC$_R$(RPW8) N-terminal domain
H1a
H1b
H2a
H2b
pre-p-loop
p-loop
Group B
partially conserved area corresponding to EDVID
H1a
H1b
H2a
H2b
Variable linker
pre-p-loop
p-loop
Two predicted β sheets present only in Group B
Group C
H1a
H1b
H2a
H2b
Variable linker
pre-p-loop
p-loop
EDVID
Group D
H1a
H1b
H2a
H2b
Variable linker
pre-p-loop
p-loop
Group E
