# Supplementary figures and images for: Genome-wide functional analyses of plant coiled–coil NLR-type pathogen receptors reveal essential roles of their N-terminal domain in oligomerization, networking, and immunity
Source: PLoS Biol. 2018 Dec 12;16(12):e2005821. doi: 10.1371/journal.pbio.2005821 (PMC6312357; doi:10.1371/journal.pbio.2005821)

**A****CC-NB-ARC**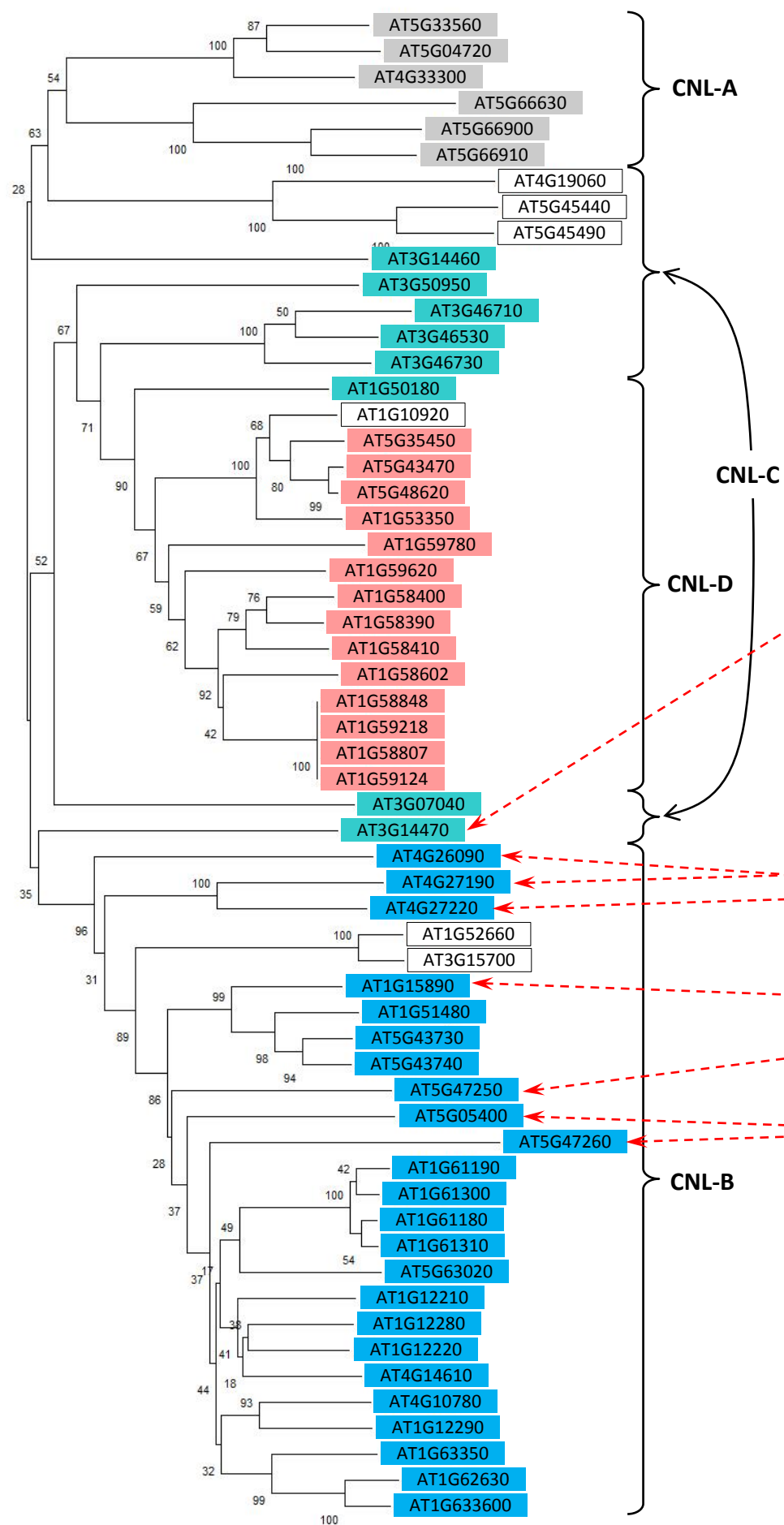**B****NB-ARC**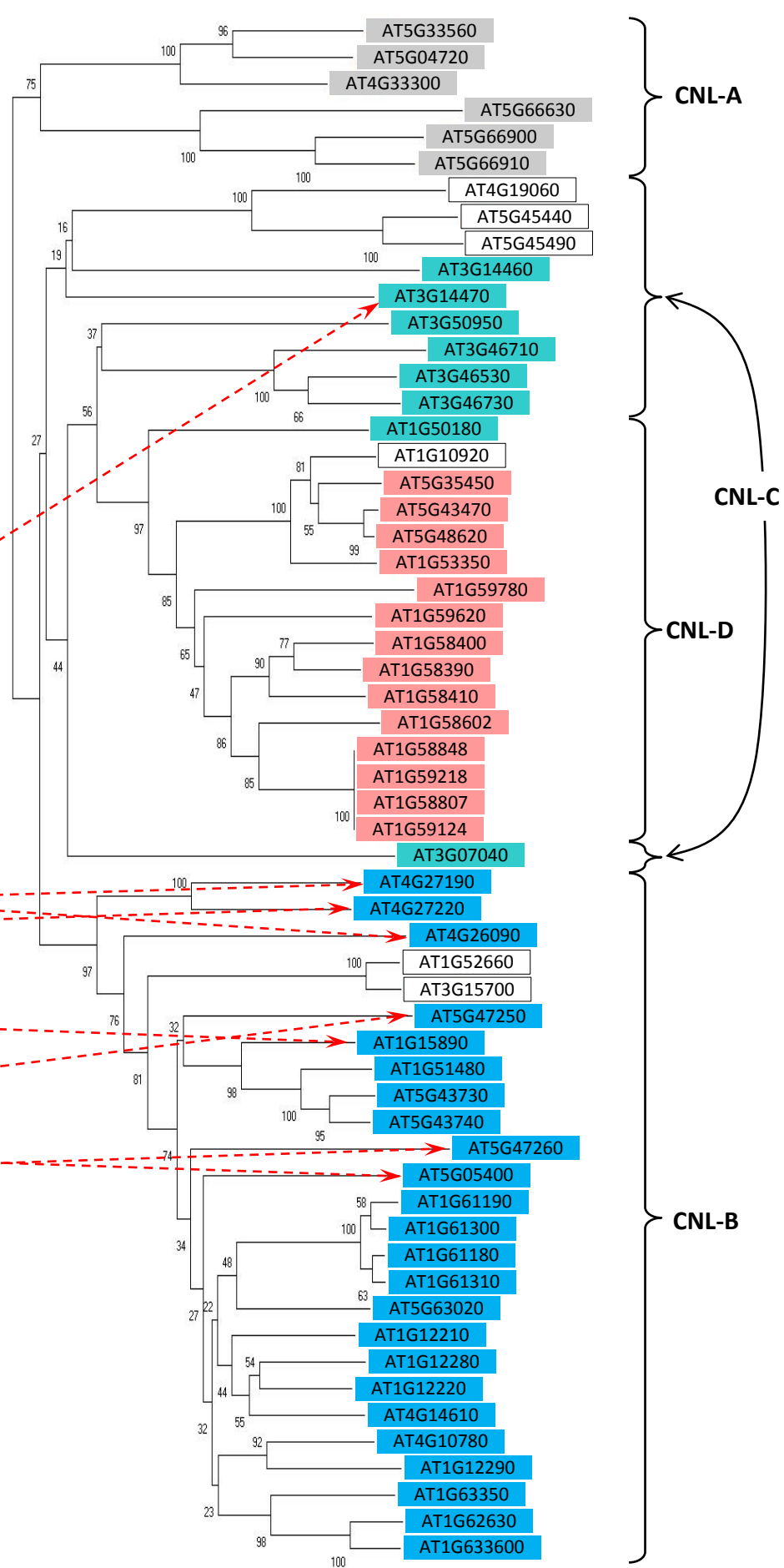

Supplement: S3 Fig — Neighbor-joining phylogenetic trees containing bootstrap values produced using either CC–NB–ARC (A) or the NB–ARC (B) sequences of CNLs in At-Col-0. The color-coded Groups identified by structure globally overlap with Groups identified previously (delineated with braces on the right of each three) based on alignment of NB–ARC domains, analysis of conserved motifs, and intron/exon distribution [17]. The dotted red lines depict clones assigned differently between the two alignments. Colors present different Groups identified by structure-based alignment of the CC domain: gray, Group A (CCR); blue, Group B; turquoise, Group C; peach, Group D; white, Group E. Color coding is the same as in Figs 2 and 4 and S2 Fig. The tree was produced using MEGA [100]. At-Col-0, Arabidopsis thaliana ecotype Columbia-0; CC, coiled–coil; CNL, CC–NLR. (PDF) [file pbio.2005821.s003.pdf]

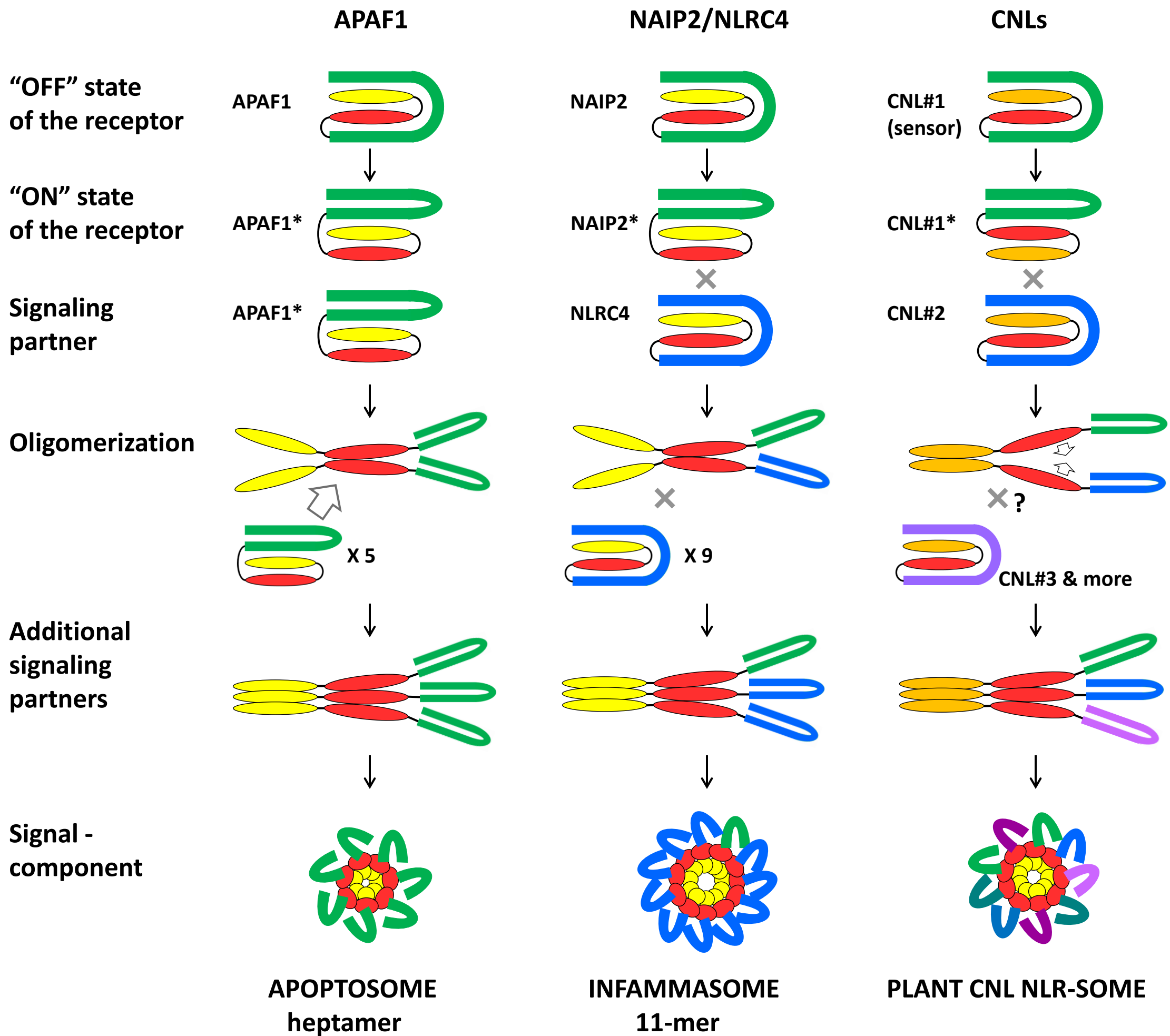

Supplement: S4 Fig — Perception of an initial signal triggers structural rearrangement to expose the NB–ARC domain in APAF1 and NOD receptors or the CC in sensor type CNLs in plants. Left: in case of APAF1, present as single isoform in the cell, the NB–ARC domain of an activated APAF1 monomer mediates oligomerization involving NB–ARC domains of additional APAF1 molecules [65]. NB–ARC–mediated oligomerization leads to the formation of a heptameric apoptosome and enables association of N-terminal CARDs and their interaction with protocaspase 9 to trigger pcd. Middle: upon activation of NAIP2, its exposed NB–ARC domain transactivates and associates with NOD receptor NLRC4 [10]. The hence exposed NB–ARC of NLRC4 triggers sequential transactivation of and associations with nine other NLRC4 monomers to produce an inflammasome composed of one NAIP2 and 10 NLRC4 subunits. BIR domains of NAIP2 and CARDs of NLRC4 interact and associate with caspases similar to CARDs in the APAF1 apoptosome. Upon activation, CC domains of CNLs in plants become exposed and trigger activation of other CNLs. Initial interactions between the monomers are likely mediated by CC domains, but similar to other STAND receptors, formation of signaling-competent complex requires interaction between the NB–ARC domains. Because the number of different CNLs may exceed several hundred in a single plant cell, other CNL receptors may also be activated and recruited to form heteromeric CNL NLRsomes. APAF1, Apoptotic Protease Activating Factor 1; BIR, Baculovirus inhibitor of apoptosis protein repeat; CARD, caspase-recruitment domain; CC, coiled–coil; CNL, CC–NLR; NAIP2, NLR family apoptosis inhibitory protein 2; NLR, nucleotide-binding leucine-rich repeat receptor; NOD, nucleotide-binding oligomerization domain; pcd, programmed cell death; STAND, signal transduction ATPases. (PDF) [file pbio.2005821.s004.pdf]
