# Supplementary material for: Genome-wide functional analyses of plant coiled–coil NLR-type pathogen receptors reveal essential roles of their N-terminal domain in oligomerization, networking, and immunity
Source: PLoS Biol. 2018 Dec 12;16(12):e2005821. doi: 10.1371/journal.pbio.2005821 (PMC6312357; doi:10.1371/journal.pbio.2005821)
Supplement: S1 File — CC, coiled–coil. (PDF) [file pbio.2005821.s005.pdf]

Molecular modeling

Homology modeling was employed for generating models of monomer CC structures of five highly interacting ECCs in group D (AT1G58390, AT1G58848, AT1G59218, AT1G58807, and AT1G5912). First step was to gather all available structures. Four structures of CNL CC domains have been solved so far from three different proteins: Rx - pdb code 4m70 [1], MLA10 - pdb codes 5t1y [2] and 3qfl [3] and wheat Sr33 - pdb code 2ncg [2]. According to the present known structures, and taking into consideration the electron density uncertainty in MLA10 3qfl, a CNL-CC monomer may adopt three possible conformations: two-helix coiled coil (mono2α), three-helix bundle (mono3α) and four-helix bundle (mono4α) represented in Figure M1.

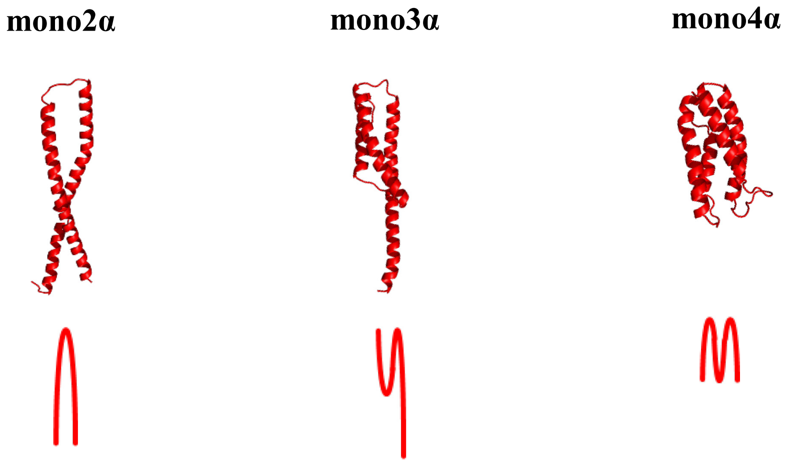

Figure M1. The three possible conformations of a CC domain monomer and their schematic representations as predicted using available structures.

These three possible structures mono 2α, 3α and 4α were further used to build monomeric 3D models of the five highly interacting ECCs in group D (AT1G58390, AT1G58848, AT1G59218, AT1G58807 and AT1G5912) based on the structure-based alignment presented in S1 Fig. The mono2α conformation is highly unlikely due to its large number of surface-exposed hydrophobic aminoacids. Also mono3α might pose problems if it doesn't meet a hydrophobic patch to attach to along the first half of the first helix. The most likely conformation of a monomer is the four-helix bundle mono4α.

Since group D AT1G59218 was shown to form homodimers by Y2H, its monomeric models were used to build all the possible dimeric configurations based on the available solved structures. Although the protein may adopt multiple configurations as a monomer or during the dimerization process, here we only atempt to describe the final dimer configuration.

To build the dimer structures, five different template configurations were generated: 2α, 3α and 4α type 1, 2 and 3 represented schematically in Figure M2. The 2α dimer consists of two intertwined 2-helix coiled coils like the MLA10-CC crystals. The 3α dimer is assembled by two

intertwined 3-helix bundle (mono3α) monomers resulting in a partial 2-helix coiled-coil complemented with additional two helices per monomer. The 4α dimers are lateral (type 1 and 2) or head-to-head (type 3) associations of mono4α. Types 1 and 2 dimer models consisted in all possible configurations that can be built using 3qfl [3] by symmetry operations at one unit cell offset from the center that, interestingly, led to extended contact interfaces between neighboring cells.

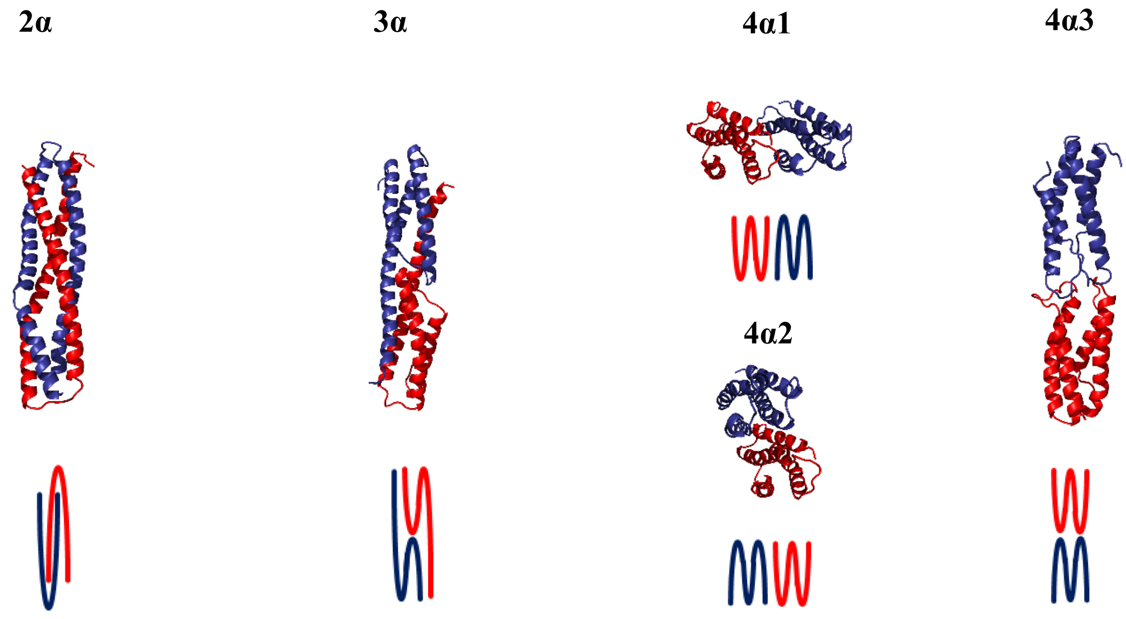

Figure M2. Five possible dimer configurations of the CC domain as derived from the available solved structures using symmetry operations and/or electron density uncertainty.

Binding free energy estimations

Based on the generated dimer templates, corresponding dimer homology models were built.
To choose the best candidate for the final equilibrium dimer configuration, binding free energies (ΔG_{bind}) were calculated employing two fundamentally different methods: molecular mechanics-based MM/PB(GB)SA method as implemented in Amber16 [4,5] and knowledge-based Prodigy method [6].
MM/PB(GB)SA calculations combine contributions from molecular mechanics internal energies (E_{MM}) and solvation free energies (ΔG_{solv}) which in turn are calculated by solving linearized Poisson Boltzman or Generalized Born equation and adding an empirically nonpolar term. These calculations are based on the thermodynamic cycle in Figure M3 and use the equation:
$\Delta G_{bind} = \Delta G_{bind,vacuum} + \Delta G_{solv,AB} - (\Delta G_{solv,A} + \Delta G_{solv,B})$,
where $\Delta G_{bind,vacuum} = \Delta E_{MM} - T\Delta S$

.

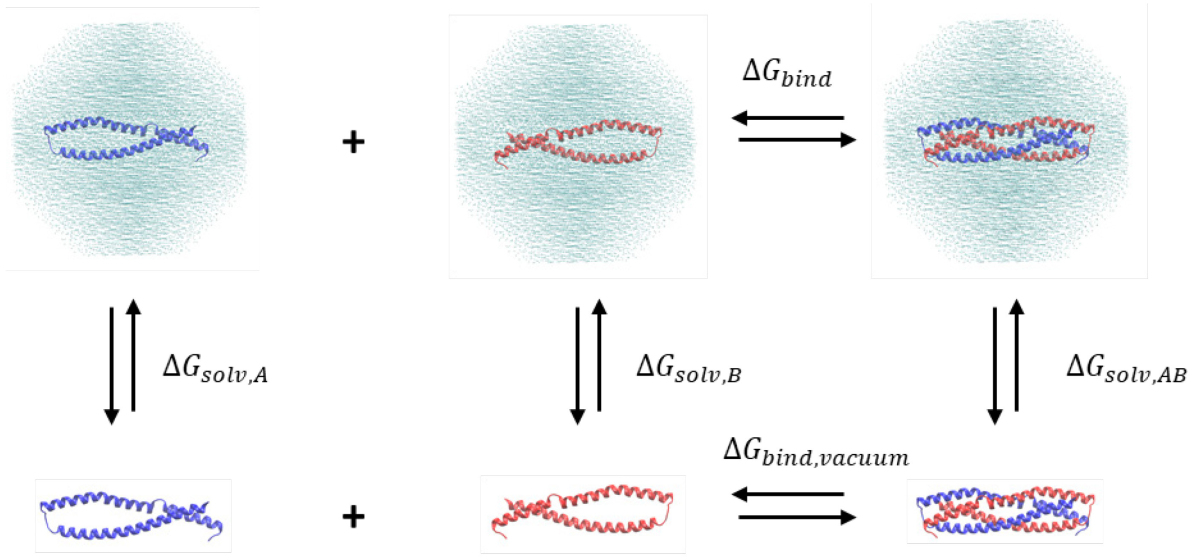

Figure M3. Thermodynamic cycle used for MM/PB(GB)SA calculations.

Based on the assumption that states of similar entropy are evaluated, the entropic contribution ($T\Delta S$) was not calculated. The results are presented in Table M1 and clearly show a lower binding free energy for the 2α.

Configuration	Prodigy ΔG (kcal/mol)	MMPBSA ΔG (kcal/mol)	MMGBSA ΔG (kcal/mol)	Prodigy kD (μM)
2α	-15.2	-19.50	-263.67	6.6e-06
3α	-10.4	-13.84	-173.18	2.3e-02
4α type 1	-5.8	24.73	-18.16	6.0e+01
4α type 2	-7.2	24.71	-25.11	5.6e+00
4α type 3	-7.4	9.01	-25.79	4.0e+00

Table M1. Estimations of binding free energies using MM/PB(GB)SA and Prodigy method, and Prodigy-derived dissociation constant.

Subsequently, MM/PB(GB)SA results were checked against the Prodigy method which uses a different reasoning. Specifically, it uses a linear regression of the number of interfacial contacts and different types of non-interacting surfaces. ΔG_{bind} calculated this way shows a strong correlation with MM/PB(GB)SA ΔG_{bind} results as shown in Figure M4C.

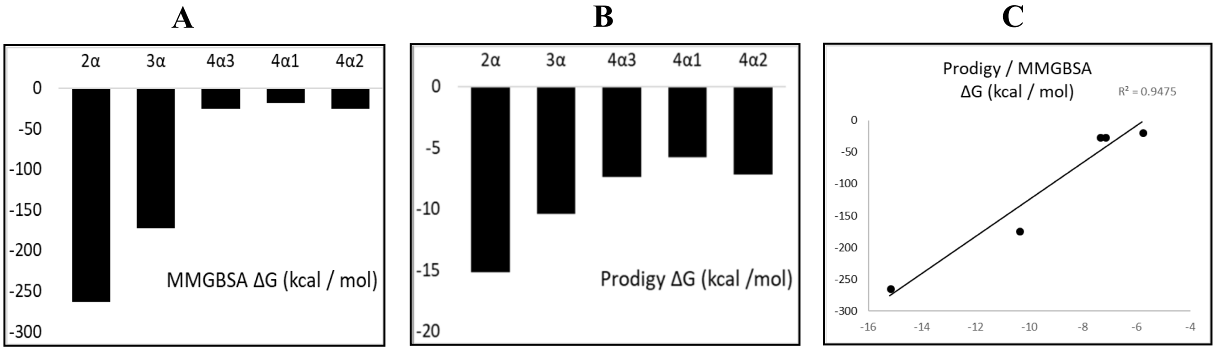

Figure M4. Binding free energies calculations using MM/GBSA (A) and Prodigy (B), and the correlation between energy values computed using both methods (C).

Dimer stability analysis

Molecular dynamics simulations were also used to asses AT1G59218 dimer stability differences among the proposed dimer configurations. For that matter, potential energy (Figure M5), root-mean-square deviations (RMSD) and root-mean-square fluctuations (RMSF) profiles were used (Figure M6).

Although the extensive in silico work suggests two intertwined helices coiled-coiled (2α) as the most probable dimer configuration, nothing is implied about CCs transitions from monomers to the dimers. It is worth mentioning though, that the flexible regions in 4α monomer become stable in the 2α dimer structure, especially the H1a-b turn (Figure. M6).

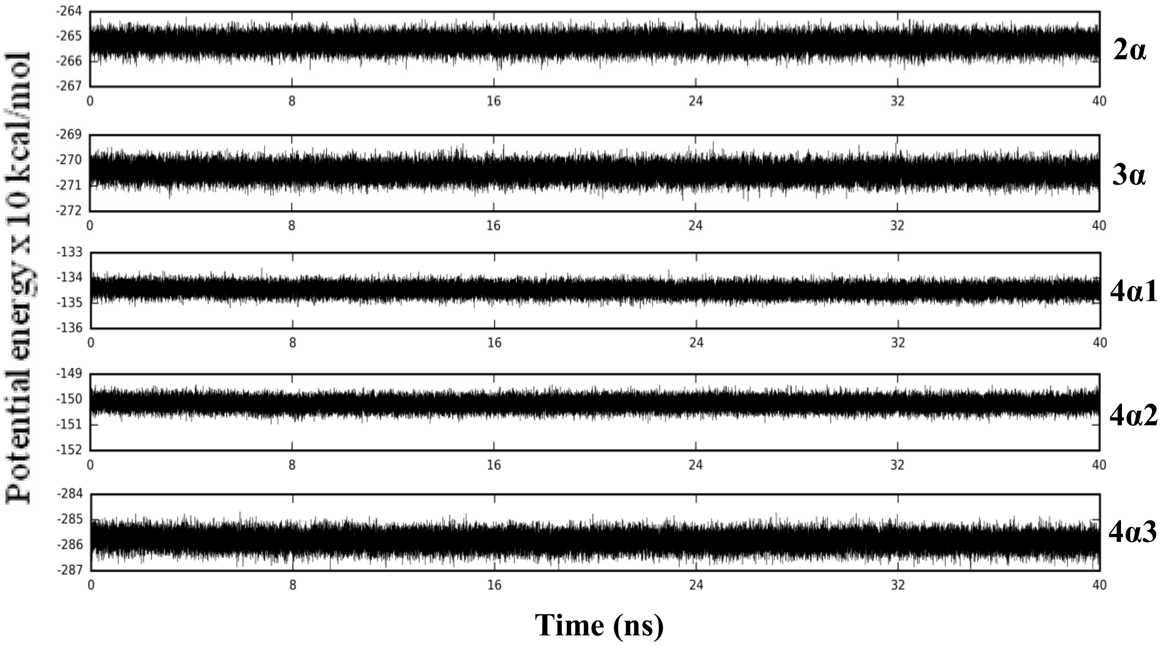

Figure M5. Molecular dynamics sanity check: Potential energy profile for 2α, 3α and 4α type 1,2,3 respectively.

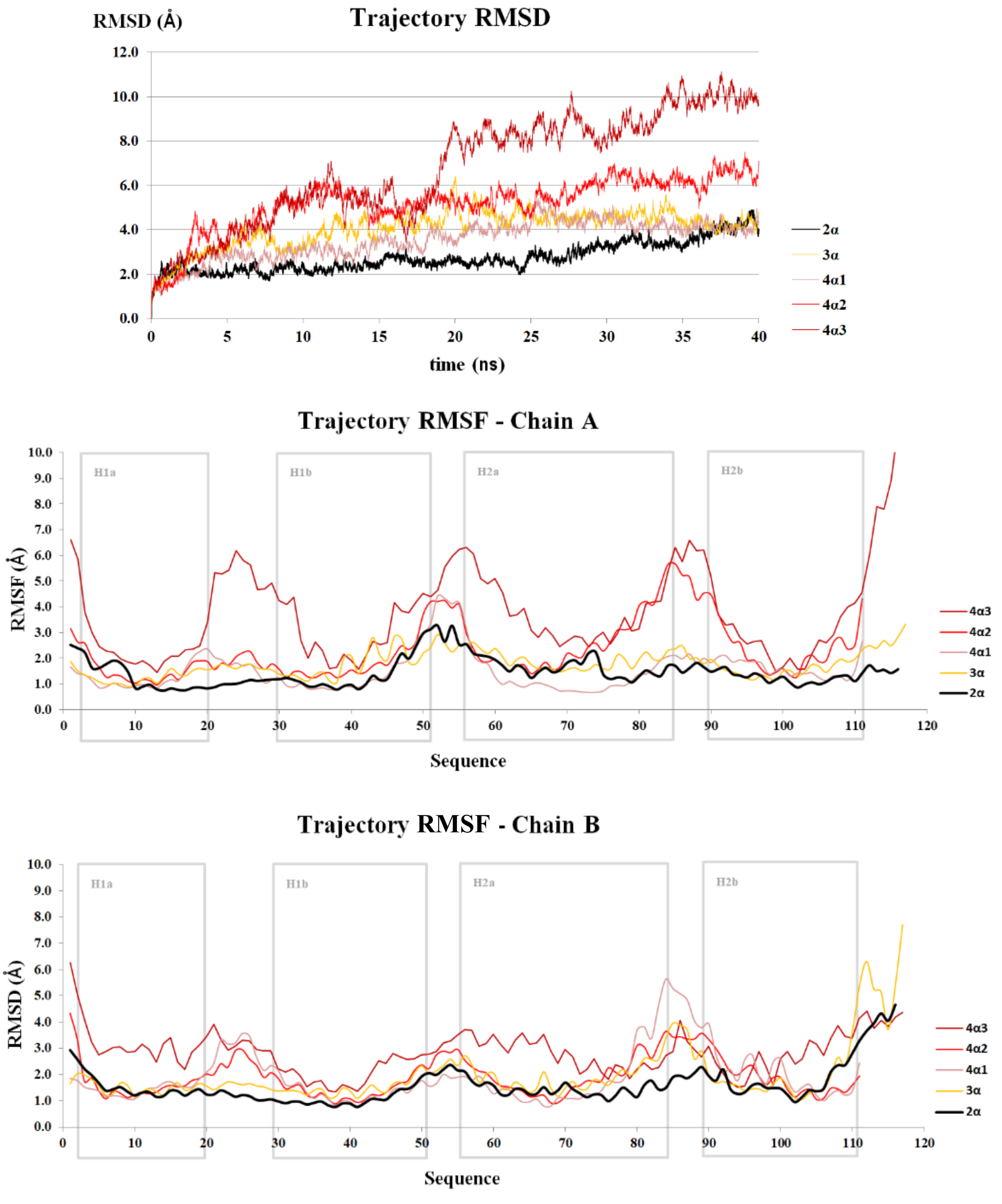

Figure M6. Molecular dynamics 40 ns simulation trajectory Cα RMSD (1st panel) and RMSF profiles (2nd and 3rd panel) of the 5 dimer conformations.

References

1. Hao W, Collier SM, Moffett P, Chai J (2013) Structural basis for the interaction between the potato virus X resistance protein (Rx) and its cofactor Ran GTPase-activating protein 2 (RanGAP2). J Biol Chem 288: 35868-35876.
2. Casey LW, Lavrencic P, Bentham AR, Cesari S, Ericsson DJ, et al. (2016) The CC domain structure from the wheat stem rust resistance protein Sr33 challenges paradigms for dimerization in plant NLR proteins. Proc Natl Acad Sci U S A 113: 12856-12861.
3. Maekawa T, Cheng W, Spiridon LN, Toller A, Lukasik E, et al. (2011) Coiled-coil domain-dependent homodimerization of intracellular barley immune receptors defines a minimal functional module for triggering cell death. Cell Host Microbe 9: 187-199.
4. Case DA, Betz RM, Cerutti DS, Cheatham TE, Darden TA, et al. (2016) AMBER 2016. University of California, San Francisco.
5. Kollman PA, Massova I, Reyes C, Kuhn B, Huo S, et al. (2000) Calculating Structures and Free Energies of Complex Molecules: Combining Molecular Mechanics and Continuum Models. Accounts of Chemical Research 33: 889-897.
6. Xue LC, Rodrigues JP, Kastritis PL, Bonvin AM, Vangone A (2016) PRODIGY: a web server for predicting the binding affinity of protein-protein complexes. Bioinformatics 32: 3676-3678.
