# Supplementary material for: Genome-wide functional analyses of plant coiled–coil NLR-type pathogen receptors reveal essential roles of their N-terminal domain in oligomerization, networking, and immunity
Source: PLoS Biol. 2018 Dec 12;16(12):e2005821. doi: 10.1371/journal.pbio.2005821 (PMC6312357; doi:10.1371/journal.pbio.2005821)
Supplement: S2 File — The fragment highlighted in gray corresponds to the ECC used in functional analyses. The P-loop is marked in red. At-Col-0, Arabidopsis thaliana ecotype Columbia-0; CNL, CC–NLR; ECC, extended CC domain. (PDF) [file pbio.2005821.s006.pdf]

Group A

AT5G66900
MNDWASLGIGSIGEAVFSKLLKVVIDEAKKFKAFKPLSKDLVSTMEILFPLTQKIDSMQKELDFG
VKELKELRDTIERADVAVRKFPRVKWYEKSKYTRKIERINKDMLKFCQIDLQLLQHRNQLTLLGL
TGNLVNSVDGLSKRMDLLSVPAPVFRDLCSVPKLDKVIVGLDWPLGELKKRLLDDSVVTLVVSA
PP**GCGKTT**
AT5G66630
MPISDVASLVGGAALGAPLSEIFKLVIEEAKKVKDFKPLSQDLASTMERLVPIFNEIDMMQQGSN
RGTSELKVLTETMERAGEMVHKCSRIQWYSIAKKALYTREIKAINQDFLKFCQIELQLIQHRNQL
QYMRSMGMASVSTKADLLSDIGNEFSKLCLVAQPEVVTKFWLKRPLMELKKMLFEDGVVTVVV
SAPY**ALGKTT**
AT5G66910
MVVVDWLGLGLGSVAGALVSEGLKVLISEAKKVLAFKSVSNELASTMESLLPVIKEIESMQDGM
ELQDLKDTIDKALLLVEKCSHVEKWNIILKSKYTRKVEEINRKMLKFCQVQLQLLLFRNQLKSMP
SMEAILNNYFQNINKKLDRLSGSPAPPLVSKRCSVPKLDNMVLVGLDWPLVELKKKLLDNSVVV
VSGPP**GCGKTT**
AT1G33560
MASFIDLFAGDITTQLLKLLALVANTVYSCKGIAERLITMIRDVQPTIREIQYSGAELSNHHQTQLG
VFYEILEKARKLCEKVLRCNRWNLKHVYHANKMKDLEKQISRFLNSQILLFVLAEVCHLRVNGD
RIERNMDRLLTERNDSLSFPETMMEIETVSDPEIQTVLELGKKKVKEMMFKFTDTHLFGISGMS
GSGKTT
AT4G33300
MAITDFFAGEIATELLKQLFTISTTAWRYKNTAKQLLTLIDSIRPTIKEIQYSGVELPAHRQAQIGM
LFDTLEKGKKLTDKVLSSKRWNLYRQLTLARKMEKLEKTISNFLKNEVFTHILADVHHLRADTSV
RLDRVDMSLDRVIQQVGSMKIGGGGLISEAMKRAEAMEIETNDDSEKFGVGLELGKVKVKKMM
FESQGGVFGISGMG**GVGKTT**
AT5G04720
MADIIGGEVVTELVRQLYAVSQKTLRCRGIAKNLATMIDGLQPTIKEIQYSGVELTPHRQAQLRM
FSETLDKCRKLTEKVLKSSRWNMVRQLLHVRKMENLQSKVSSFLNGQLLVHVLADVHHVRAD
SEFRFDRIDRKVDSLNEKLGSMKLRGSESLREALKTAEATVEMVTTDGADLGVGLDLGKRKVK
EMLFKSIDGERLIGISGMS**GSGKTT**

Group B

AT4G27220
MFRSNARALNRALERLKNVQTKVNEALKRSGIQEKSLERKLRIWLRKVEENVPLGELILEKRSS
CAIWLSDKDVEILEKVKRLEEQGQDLIKKISVNKSSREIVERVLGPSFHPQKTALEMLDKLKDCL
KKKNVQKIGVWGMG**GVGKTT**
AT4G27190
MECCAPVIGEILRLMYESTFSRVANAIKFKSNVKALNESLERLTELKGNMSEDHETLLTKDKPLR
LKLMRWQREAEEVISKARLKLEERVSCGMSLRPRMSRKLVKILDEVKMLEKDGIEFVDMLSVES
TPERVEHVPGVSVVHQTMASNMLAKIRDGLTSEKAQKIGVWGMG**GVGKTT**

AT4G26090
MDFISSLIVGCAQVLCESMNMAERRGHKTDLRQAITDLETAIGDLKAIRDDLTLRIQQDGLEGRS
CSNRAREWLSAVQVTETKTALLLVRFRRREQRTRMRRRYLSCFGCADYKLCKKVSAILKSIGEL
RERSEAIKTDGGSIQVTCREIPIKSVVGNTTMMEQVLEFLSEEEERGIIGVYGPG**GVGKTT**
AT5G47250

MNCCWQVVEPCYKSALSYLCVKVGNICMLKENLVLLKSAFDELKAEKEDVVNRVNAGELKGG
QRLAIVATWLSQVEIIEENTKQLMDVASARDASSQNASAVRRRLSTSGCWFSTCNLGEKVFKK
LTEVKSLSGKDFQEVTEQPPPPVVEVRLCQQTVGLDTTLEKTWESLRKDENRMLGIFGMG**GV**
GKTT
AT5G47260
MGNNFSVESPSLAPFLCGKRKYLYNLERNLEALHKVMQDLNAMRNDLLKRLSKEEEIGLQGLQ
EVKEWISMVEEIEPKANRLLDESVSEIQRLSRYGYCSLIPASTYRYSEKVLTTMEGVETLRSKGV
FEAVVHRALPPLVIKMPPIQLTVSQAKLLDTAWARLMDINVGTLGIYGRG**GVGKTT**
AT5G05400
MGACFSVAISCDQAVNNLTSCLSRNQNRFRNLVDHVAALKKTVRQLEARRDDLLKRIKVQEDR
GLNLLDEVQQWLSEVESRVCEAHDILSQSDEEIDNLCCGQYCSKRCKYSYDYSKSVINKLQDV
ENLLSKGVFDEVAQKGPIPKVEERLFHQEIVGQEAIVESTWNSMMEVGVGLLGIYGMG**GVGKT**
T
AT1G15890
MGNCVALEISCDQTLNHACGCLFGDRNYILKMEANLEALQNTMQELEERRDDLLRRVVIEEDK
GLQRLAQVQGWLSRVKDVCSQVNDLLKAKSIQTERLCLCGYCSKNFISGRNYGINVLKKLKHV
EGLLAKGVFEVVAEKIPAPKVEKKHIQTTVGLDAMVGRAWNSLMKDERRTLGLYGMG**GVGKT**
T
AT1G51480 (AT1G51485)
MVQHWDGDPAVRTKSHIEMLHPACEMSWNLKQHDDTAASLMVIQSPTSDPVFNPVHTIKQFG
ERYTQDFVIRERDKSFGVLIHCFCKMADWLLLIPWNKIFTAACGCFFSDRNYIHKMEANLDDLH
TTMEELKNGRDDLLRRVSIEEDKGLQQLAQVKGWISRVEIVESRFKDLLEDKSTETGRLCLFGF
CSENCISSYNYGEKVMKNLEEVKELLSKKHFEVVAHKIPVPKVEEKNIHTTVGLYAMVEMAWKS
LMNDEIRTLCLHGMG**GVGKTT**
AT5G43740
MLGWLVIPWNQIFTAACGCFLSDRNYIHMMESNLDALQKTMEELKNGRDDLLGRVSIEEDKGL
QRLAQVNGWLSRVQIVESEFKDLLEAMSIETGRLCLLGYCSEDCISSYNYGEKVSKMLEEVKEL
LSKKDFRMVAQEIIHKVEKKLIQTTVGLDKLVEMAWSSLMNDEIGTLGLYGMG**GVGKTT**
AT5G43730
MVDWLSLLPWNKIFTAACGCFLSDSNYIHLMESNLDALQKTMEELKNGRDDLLARVSIEEDKGL
QRLALVNGWLSRVQIVESEFKDLLEAMSIETGRLCLFGYCSEDCISSYNYGGKVMKNLEEVKEL
LSKKNFEVVAQKIIPKAEKKHIQTTVGLDTMVGIAWESLIDDEIRTLGLYGMG**GIGKTT**
AT5G63020
MGGCVSVSISCDQLTKNVCSCLNRNGDYIHGLEENLTALQRALEQIEQRREDLLRKILSEERRG
LQRLSVVQGWVSKVEAIVPRVNELVRMRSVQVQRLCLCGFCSKNLVSSYRYGKRVMKMIEEV
EVLRYQGDFAVVAERVDAARVEERPTRPMVAMDPMLESAWNRLMEDEIGILGLHGMG**GVGK**
TT
AT1G61190
MGNFVCIEISGDQMLDRIIRCLCGKGYIRNLEKNLRALQREMEDLRATQHEVQNKVAREESRH
QQRLEAVQVWLDRVNSIDIECKDLLSVSPVELQKLCLCGLCSKYVCSSYKYGKRVFLLLEEVTK
LKSEGNFDEVSQPPPRSEVEERPTQPTIGQEEMLKKAWNRLMEDGVGIMGLHGMG**GVGKTT**
AT1G61180
MGSCFSLQVSDQTLNRIFNCLIGKSYIRTLEKNLRALQREMEDLRAIQHEVQNKVARDEARHQR
RLEAVQVWLDRVNSVDIECKDLLSVTPVELQKLCLCGLCSKYVCSSYKYGKKVFLLLEEVKKLN
SEGNFDEVSQPPPRSEVEERPTQPTIGQEDMLEKAWNRLMEDGVGIMGLHGMG**GVGKTT**
AT1G61310
MGSCFSFQIAVGDQTMNRIFDCLIGKSYIRTLEQNLRALQREMEDLRATQHEVQNKVAREESR
HQQRLEAVQVWLDRVNSIDIECKDLLSVSPVELQKLCLCGLCTKYVCSSYKYGKKVFLLLEEVKI
LKSEGNFDEVSQPPPRSEVEERPTQPTIGQEEMLEKAWNRLMEDGVGIMGLHGMG**GVGKTT**
AT1G61300

MGCCFSVQFSFDDQTLVRIFNFLCGNINRNSFGVEERPTQPTIGQEEMLEKAWNRLMEDRVGI
MGLHGMG**GVGKTT**
AT1G63350
MGISFSIPFDPCVNKVSQWLDMKVSYTHNLEKNLVALETTMEELKAKRDDLLRKLKREEDRGL
QTLGEIKVWLNRVETIESRVNDLLNARNAELQRLCLCGFCSKSLTTSYRYGKSVFLKLREVEKL
ERRVFEVISDQASTSEVEEQQLQPTIVGQETMLDNAWNHLMEDGVGIMGLYGMG**GVGKTT**
AT1G62630
MGISFSIPFDPCVNKVSQWLDMKGSYTHNLEKNLVALETTMEELKAKRDDLLRRLKREEDRGL
QRLSEFQVWLNRVATVEDIIITLLRDRDVEIQRLCLCRFCSKNLTTSYRYGKSVFLRLREVEKLK
GEVFGVITEQASTSAFEERPLQPTIVGQKKMLDKAWKHLMEDGTGIMGMYGMG**GVGKTT**
AT1G63360
MGISFSIPFDPCVNKVSQWLDMKVSYTHNLEKNLAALEKTMKELKAKRDDLERRLKREEARGL
QRLSEFQVWLDSVATVEDIIITLLRDRNVEIQRLCLCRFCSKSLTRSYRYGKSVFLRLREVEKLK
GEVFGVITEQASTSAFEERPLQPTIVGQDTMLDKAGKHLMEDGVGIMGMYGMG**GVGKTT**
AT1G12280
MGACLTLSFSCDEVVNQISQGLCINVGYICELSKNVVAMKKDMEVLKKKRDDVKRRVDIEEFTR
RRERLSQVQGWLTNVSTVENKFNELLTTNDAELQRLCLFGFCSKNVKMSYLYGKRVVLMLKEI
ESLSSQGDFDTVTLATPIARIEEMPIQPTIVGQETMLERVWTRLTEDGDEIVGLYGMG**GVGKTT**
AT4G14610
MGGCISVSVSCDQFVNQFSQWLCVRKGYIHSLPENLAALQKAIEVLKTKHDDVKRRVDKEEFL
GRRHRLSQVQVWLTNVVIIEKRFNDLFSNKEVEIERLCFCGFCSKSFGKSYHYGKMVSVMLKE
VENLSSRGVFDVVTEENLVAQVEEMPIQSTVVGQETMLERVWNTLMKDGFKIMGLYGMG**GVG
KTT**
AT1G12210
MGGCVSVSLSCDREVNQFSQWLCVSGSYIQNLSENLASLQKAMGVLNAKRDDVQGRINREEF
TGHRRRLAQVQVWLTRIQTIENQFNDLLSTCNAEIQRLCLCGFCSKNVKMSYLYGKRVIVLLRE
VEGLSSQGVFDIVTEAAPIAEVEELPIQSTIVGQDSMLDKVWNCLMEDKVWIVGLYGMG**GVGK
TT**
AT1G12220
MGGCFSVSLPCDQVVSQFSQLLCVRGSYIHNLSKNLASLQKAMRMLKARQYDVIRRLETEEFT
GRQQRLSQVQVWLTSVLIIQNQFNDLLRSNEVELQRLCLCGFCSKDLKLSYRYGKRVIMMLKE
VESLSSQGFFDVVSEATPFADVDEIPFQPTIVGQEIMLEKAWNRLMEDGSGILGLYGMG**GVGK
TT**
AT4G10780
MGSCISLQISCDQVLTRAYSCFFSLGNYIHKLKDNIVALEKAIEDLTATRDDVLRRVQMEEGKGL
ERLQQVQVWLKRVEIIRNQFYDLLSARNIEIQRLCFYSNCSTNLSSSYTYGQRVFLMIKEVENLN
SNGFFEIVAAPAPKLEMRPIQPTIMGRETIFQRAWNRLMDDGVGTMGLYGMG**GVGKTT**
AT1G12290
MGGCVSVQVSCDQLLNHLGRCFCRKLYYIQNIKENLTSLEEAMEDLKALRDDLLRKVQTAEEG
GLQRLHQIKVWLKRVKTIESQFNDLDSSRTVELQRLCCCGVGSRNLRLSYDYGRRVFLMLNIVE
DLKSKGIFEEVAHPATRAVGEERPLQPTIVGQETILEKAWDHLMDDGTKIMGLYGMG**GVGKTT**

Group C

AT3G46710
MVDAITEFVVGKIDNYLIEEAPMLIGVKDDLEELKTELTCIQVYLKNVEVCDKEDEVSKEWTKLVL
DIAYDVEDVLDTYFLKLEKRLHRLGLMRLTNIISDKKDAYNILDDIKTLKRRTLDVTRKLEMYGIGN

FNEHRVVASTSRVREVRRARSDDQEERVVGLTDDAKVLLTKLLDDDGDNKIYMISIFGME**GLG
KTS**
AT3G46530
MVDAITEFVVGKIGNYLIEEASMFMAVKEDLEELKTELTCIHGYLKDVEAREREDEVSKEWSKLV
LDFAYDVEDVLDTYHLKLEERSQRRGLRRLTNKIGRKMDAYSIVDDIRILKRRILDITRKRETYGI
GGLKEPQGGGNTSSLRVRQLRRARSVDQEEVVVGLEDDAKILLEKLLDYEEKNRFIISIFGMG**G
LGKTA**
AT3G46730
MVDAVTGFVLNKIGGYLINEVLALMGVKDDLEELKTELTCIHGYLKDVEAREREDEVSKEWTKL
VLDIAYDIEDVLDTYFLKLEERSLRRGLLRLTNKIGKKRDAYNIVEDIRTLKRRILDITRKRETFGIG
SFNEPRGENITNVRVRQLRRAPPVDQEELVVGLEDDVKILLVKLLSDNEKDKSYIISIFGMG**GLG
KTA**
AT1G50180
MAEAIVSVTVQKLGQLLLEEPLFLFGIGDQVKQLQDELKRLNCFLKDADEKQHESERVRNWVA
GIREASYDAEDILEAFFLKAESRKQKGMKRVLRRLACILNEAVSLHSVGSEIREITSRLSKIAASM
LDFGIKESMGREGLSLSDSLREQRQSFPYVVEHNLVGLEQSLEKLVNDLVSGGEKLRVTSICG
MG**GLGKTT**
AT3G50950
MVDAVVTVFLEKTLNILEEKGRTVSDYRKQLEDLQSELKYMQSFLKDAERQKRTNETLRTLVAD
LRELVYEAEDILVDCQLADGDDGNEQRSSNAWLSRLHPARVPLQYKKSKRLQEINERITKIKSQ
VEPYFEFITPSNVGRDNGTDRWSSPVYDHTQVVGLEGDKRKIKEWLFRSNDSQLLIMAFVGMG
GLGKTT
AT3G07040
MASATVDFGIGRILSVLENETLLLSGVHGEIDKMKKELLIMKSFLEDTHKHGGNGSTTTTTQLFQ
TFVANTRDLAYQIEDILDEFGYHIHGYRSCAKIWRAFHFPRYMWARHSIAQKLGMVNVMIQSIS
DSMKRYYHSENYQAALLPPIDDGDAKWVNNISESSLFFSENSLVGIDAPKGKLIGRLLSPEPQRI
VVAVVGMG**GSGKTT**
AT3G14460
MANSYLSSCANVMVERINTSQELVELCKGKSSSALLKRLKVALVTANPVLADADQRAEHVREV
KHWLTGIKDAFFQAEDILDELQTEALRRRVVAEAGGLGGLFQNLMAGREAIQKKIEPKMEKVVR
LLEHHVKHIEVIGLKEYSETREPQWRQASRSRPDDLPQGRLVGRVEDKLALVNLLLSDDEISIGK
PAVISVVGMP**GVGKTT**
AT3G14470
MTGIGEMFLAAFLQALFQTLVSEPFRSFFKRRELNENLLERLSTALLTITAVLIDAEEKQITNPVV
EKWVNELRDVVYHAEDALDDIATEALRLNIGAESSSSNRLRQLRGRMSLGDFLDGNSEHLETR
LEKVTIRLERLASQRNILGLKELTAMIPKQRLPTTSLVDESEVFGRDDDKDEIMRFLIPENGKDN
GITVVAIVGIG**GVGKTT**

Group D

AT1G59620
MAETLLSFGVEKLWDLLVRESDRFQGVKKQFNELRSDLNKLRCFLEDADAKKHQSAMVSNTV
KEVKEIVYDTEDIIETFLRKKQLGRTRGMKKRIKEFACVLPDRRKIAIDMEGLSKRIAKVICDMQS
LGVQQVIVNDEYMQSLQERQKXNMRQTFSNNNESVLVGLEENVKKLVGHLVEVEDSSQVVSIT
GMG**GIGKTT**
AT1G53350
MAEAVVSFGVEKLWELLSRESARLNGIDEQVDGLKRQLGRLQSLLKDADAKKNETERVRNFLE
DVKDIVYDADDIIESFLLNELRGKEKGIKKQVRTLACFLVDRRKFASDIEGITKRISEVIVGMQSLG
IQHIADGGGRSLSLQERQREIRQTFSRNSESDLVGLDQSVEELVDHLVENDSVQVVSVSGMG**G
IGKTT**
AT5G35450

MAEGVVSFGVQKLWALLNRESERLNGIDEQVDGLKRQLRGLQSLLKDADAKKHGSDRVRNFL
EDVKDLVFDAEDIIESYVLNKLRGEGKGVKNHVRRLACFLTDRHKVASDIEGITKRISKVIGEMQ
SLGIQQQIIDGGRSLSLQDIQREIRQTFPNSSESDLVGVEQSVEELVGPMVEIDNIQVVSISGMG
GIGKTT
AT5G43470
MAEAFVSFGLEKLWDLLSRESERLQGIDGQLDGLKRQLRSLQSLLKDADAKKHGSDRVRNFLE
DVKDLVFDAEDIIESYVLNKLSGKGKGVKKHVRRLACFLTDRHKVASDIEGITKRISEVIGEMQSF
GIQQIIDGGRSLSLQERQRVQREIRQTYPDSSESDLVGVEQSVKELVGHLVENDVHQVVSIAGM
G**GIGKTT**
AT5G48620
MAEGFVSFGLEKLWDLLSRESERLQGIDEQLDGLKRQLRSLQSLLKDADAKKHGSDRVRNFLE
DVKDLVFDAEDIIESYVLNKLRGEGKGVKKHVRRLARFLTDRHKVASDIEGITKRISDVIGEMQS
FGIQQIIDGVRSLSLQERQRVQREIRQTYPDSSESDLVGVEQSVEELVGHLVENDIYQVVSIAG
MG**GIGKTT**
AT1G59780
MQDLYMVDSIVSFGVEKLWKLLSQEYERFQGVEEQITELRDDLKMLMAFLSDADAKKQTRALA
RNCLEEIKEITYDAEDIIEIFLLKGSVNMRSLACFPGGRREIALQITSISKRISKVIQVMQNLGIKSDI
MDGVDSHAQLERKRELRHTFSSESESNLVGLEKNVEKLVEELVGNDSSHGVSITGLG**GLGKTT**
AT1G58400
MVEAIVSFGVEKLWDRLTQEYEQFQGVEDRIAELKSNLNLLKSFLKDAEAKKNTSQMVRHCVE
EIKEIVYDTENMIETFILKEAARKRSGIIRRITKLTCIKVHRWEFASDIGGISKRISKVIQDMHSFGV
QQMISDGSQSSHLLQEREREMRQTFSRGYESDFVGLEVNVKKLVGYLVEEDDIQIVSVTGMG
GLGKTT
AT1G58410
MELVSFGVEKLWDRLSQEYDQFKGVEDQVTELKSNLNLLKSFLKDADAKKHISEMVRHCVEEI
KDIVYDTEDIIETFILKEKVEMKRGIMKRIKRFASTIMDRRELASDIGGISKRISKVIQDMQSFGVQ
QIITDGSRSSHPLQERQREMRHTFSRDSENDFVGMEANVKKLVGYLVEKDDYQIVSLTGMG**GL
GKTT**
AT1G58602
MAGELVSFAVNKLWDLLSHEYTLFQGVEDQVAELKSDLNLLKSFLKDADAKKHTSALVRYCVE
EIKDIVYDAEDVLETFVQKEKLGTTSGIRKHIKRLTCIVPDRREIALYIGHVSKRITRVIRDMQSFG
VQQMIVDDYMHPLRNREREIRRTFPKDNESGFVALEENVKKLVGYFVEEDNYQVVSITGMG**GL
GKTT**
AT1G58390
MAGELVSFGIKKLWDLLSQECEQFQGVEDQVTGLKRDLNLLSSFLKDADAKKHTTAVVRNVVE
EIKEIVYDAEDIIETYLLKEKLWKTSGIKMRIRRHACIISDRRRNALDVGGIRTRISDVIRDMQSFG
VQQAIVDGGYMQPQGDRQREMRQTFSKDYESDFVGLEVNVKKLVGYLVDEENVQVVSITGM
G**GLGKTT**
AT1G58848 (AT1G58842)
MAGELISFGIQNLWNLLSQECELFQGVEDQVTELKRDLNLLSSFLKDADAKKHTSAVVKNCVEE
IKEIIYDGEDTIETFVLEQNLGKTSGIKKSIRRLACIIPDRRRYALGIGGLSNRISKVIRDMQSFGVQ
QAIVDGGYKQPQGDKQREMRPRFSKDDDSDFVGLEANVKKLVGYLVDEANVQVVSITGMG**GL
GKTT**
AT1G59218
MAGELISFGIQNLWNLLSQECELFQGVEDQVTELKRDLNLLSSFLKDADAKKHTSAVVKNCVEE
IKEIIYDGEDTIETFVLEQNLGKTSGIKKSIRRLACIIPDRRRYALGIGGLSNRISKVIRDMQSFGVQ
QAIVDGGYKQPQGDKQREMRPRFSKDDDSDFVGLEANVKKLVGYLVDEANVQVVSITGMG**GL
GKTT**
AT1G59124
MAGELISFGIQNLWNLLSQECELFQGVEDQVTELKRDLNMLSSFLKDANAKKHTSAVVKNCVE
EIKEIIYDGEDTIETFVLEQNLGKTSGIKKSIRRLACIIPDRRRYALGIGGLSNRISKVIRDMQSFGV

QQAIVDGGYKQPQGDKQREMRQKFSKDDDSDFVGLEANVKKLVGYLVDEANVQVVSITGMG
GLGKTT
AT1G58807
MAGELISFGIQNLWNLLSQECELFQGVEDQVTELKRDLNMLSSFLKDANAKKHTSAVVKNCVE
EIKEIIYDGEDTIETFVLEQNLGKTSGIKKSIRRLACIIPDRRRYALGIGGLSNRISKVIRDMQSFGV
QQAIVDGGYKQPQGDKQREMRQKFSKDDDSDFVGLEANVKKLVGYLVDEANVQVVSITGMG
GLGKTT

Group E

AT3G15700
MGKDFKSMVTRCIYVGKENDNVKKLKTATEELKDLRNIVMKRVKMYEDQQKLKRLEKVQVWL
RQADVAIKEAEEMLITLMSSSSSNGSSMMSFHKLDKKLCKKLKEVQEIKSRGTFDVVVENSGIG
SGSMMISNVDRDDQTVGLEAVSGLVWRCMTVDNTGIIGLYGVE**GVGKTT**
AT1G52660
MGKDFKSLVTRCIYVGKMNDNAKKLKIATEELKDLGNNVMKRVKLCEEQQQMKRLDKVQTWL
RQADTVIKEAEEYFLMSSSSSSSGLISSSHKMEKKICKKLKEVQEIKSRGMFEVVAESTGGIGG
GAGGGLTIKDSDEQTIGLEAVSGLVWRCLTMENTGIIGLYGVE**GVGKTT**
AT5G45440
MTQEDSSRGLTSVGRVDFTNRFADRYNEWLGTTGDETKQVEDRVETDSGLPGHDIYGFENEI
KSLQHFLLDQKSYKLFKSLVVVGEY**GVGKTA**
AT5G45490
MPSKNLQQAVLTNEFTTNFITTCKDWLDVNGLAKGNLEKKRDDNEEEERLKTESKLPGHDIHG
FDNEIKSLQHFLLDQKVRREFKSLVIVGEY**GVGKTA**
AT4G19060
MDIAKKFISEIDDKLESKSEFDKELEKIKSSFNEEYEKWSSGKQRGSSSKHGNQSTHGDSSPTR
NSSGSSKKGRPKANRVETSSELPDHLIRGFINEKLFLKNFLLKQKESEEFKTLAIVGKY**GVGKTT**
AT1G10920
MKSLGIQEIIDGASSMSLQERQREQKEIRQTFANSSESDLVGVEQSVEALAGHLVENDNIQVVSI
SGMG**GIGKTT**
