# Supplementary material for: Genome-wide functional analyses of plant coiled–coil NLR-type pathogen receptors reveal essential roles of their N-terminal domain in oligomerization, networking, and immunity
Source: PLoS Biol. 2018 Dec 12;16(12):e2005821. doi: 10.1371/journal.pbio.2005821 (PMC6312357; doi:10.1371/journal.pbio.2005821)
Supplement: S3 File — Avr, avirulence factor; ECC, extended CC domain; HR, hypersensitive response; KO, knockout. (PDF) [file pbio.2005821.s007.pdf]

Immune trigger	CNL	Partner CNL	KO accession (TAIR)	Phenotype compared to Col-0	Remark
AvrB$_{Pgy}$	AT3G07040	AT4G14610	SALK_080562	no change	AT3G07040-ECC expression does not induce necrosis.<br>Activation of AT3G07040 by AvrB triggers HR[1] in absence and presence of AT1G14610.
ECC	AT3G46710	AT1G61310<br>AT4G10780	SALK_125189<br>SALK_066132	no change<br>not tested	AT3G46710-ECC interacts with ECCs-AT1G61310 and -AT4G10780. No homozygous SALK_066132 obtained, possible lethal.
ECC	AT1G51480	AT5G43740<br><br><br>AT5G45440	SALK_025605<br>SALK_031303<br>SALK_128856<br>SALK_023316	no change<br>no change<br>no change<br>no change	AT1G51480-ECC interacts with ECCs of AT5G43740 and AT5G45440. Both are frequent interactors and potential hubs.
ECC	AT1G12210	AT1G61310<br>AT1G12220	SALK_125189<br>CS807128<br>CS92107	no change<br>no change<br>no change	AT1G12210-ECC interacts with ECCs of AT1G61310 and AT1G12220. Both are frequent interactors and potential hubs

Additional note: According to the sensor/actor model sensors are not capable to autonomously signal while actors are. This model is consistent with the profound necrosis induced upon CCRs expression that was also observed in our study. Also accordingly, ECC corresponding to putative sensors (e.g. Group D) did not trigger plant responses. Therefore these sensors cannot be used to test the existence of a network as these assays rely on a phenotypic readout. For instance, the ECCs corresponding to the R genes *RPM1* (AT3G07040) and *ZAR1*(AT3G50950), act genetically as sensors and did not induce necrosis in our system. To overcome this we tested if RPM1-mediated necrosis induced by AvrB$_{Pgy}$ persisted in a knockout of RPM1-ECC interactor AT1G14610-ECC. No phenotypic change was observed, which is indicative of redundancy and the presence of an additional partner that was not identified in Y2H. ECC of ZAR1 has three interactors, two of which are ECC's of ADR homologs (Fig 2B) and the third one corresponds to AT4G10780. We know from personal communication with groups working on ZAR1, that ZAR1 is not dependent on ADRs. Our attempts to produce a homozygous T-DNA insertion for the third interactor, AT4G10780, failed. This prevented us from validation of the dependence of ZAR1 on its interactors. As listed above we also expressed three different HR-inducing ECC in Arabidopsis mutants in which their interactors were knocked out. However, for none of them a phenotypic change was observed, again indicating redundancy in signaling. Most necrosis-inducing clones have many interactors, which makes it practically impossible to pyramid knockouts of these interactors in a single line. Together these data imply that mutation of one or more interacting CNLs does not compromise the signaling of the ECC, supporting the idea of a network in which each CNL can signal via (multiple) other CNLs.

1. Bisgrove SR, Simonich MT, Smith NM, Sattler A, Innes RW (1994) A disease resistance gene in Arabidopsis with specificity for two different pathogen avirulence genes. Plant Cell 6: 927-933.
