# Supplementary material for: Genome-wide functional analyses of plant coiled–coil NLR-type pathogen receptors reveal essential roles of their N-terminal domain in oligomerization, networking, and immunity
Source: PLoS Biol. 2018 Dec 12;16(12):e2005821. doi: 10.1371/journal.pbio.2005821 (PMC6312357; doi:10.1371/journal.pbio.2005821)
Supplement: S4 File — ECC, extended CC domain. (PDF) [file pbio.2005821.s008.pdf]

I. Swap mutants

AT1G63360

```
MGISFSIPFDPCVNKVSQWLDMKVSYTHNLEKNLAALEKTMKELKAKRDDLERRLKREEARGLQRLSEFQVWLDSVATVEDIIITLLRD
RNVEIQRLCLCRFCSKSLTRSYRYGKSVFLRLREVEKLKGEVFGVITEQASTSAFEERPLQPTIVGQDTMLDKAGKHLMEDG
```

AT1G15890

```
MGNCVALEISCDQTLNHACGCLFGDRNYILKMEANLEALQNTMQELEERRDDLLRRVVIEEDKGLQRLAQVQGWLSRVKDVCSQVNDLL
KAKSIQTERLCLCGYCSKNFISGRNYGINVLKKLKHVEGLLAKGVFEVVAEKIPAPKVEKKHIQTTVGLDAMVGRAWNSLMKDER
```

```
A1          MG--ISFSIPFDPCVNKVSQWLDMKVSYTHNLEKNLAALEKTMKELKAKRDDLERRLKRE
A2          MGNCVALEISCDQTLNHACGCLFGDRNYILKMEANLEALQNTMQELEERRDDLLRRVVIE
            **  :::.*  *  :*:..  *  . .*  ::* ** **::**:**: :**** **:  *

A1          EARGLQRLSEFQVWLDSVATVEDIIITLLRDRNVEIQRLCLCRFCSKSLTRSYRYGKSVF
A2          EDKGLQRLAQVQGWLSRVKDVCSQVNDLLKAKSIQTERLCLCGYCSKNFISGRNYGINVL
            * :*****::.* **. *  * . :  **: :.:: :***** :***.:  . .** .*:

A1          LRLREVEKLLAKGVFEVVAEKIPAPKVEKKHI-QTTVGLDAMVGRAWNSLMKDER
A2          KKLKHVEGLK-GEVFGVITEQASTSAFEERPLQPTIVGQDTMLDKAGKHLMEDG
             :*:.** *    ** *::*:  :  .*::  :  * ** *:*: :* : **:*


A3          MG--ISFSIPFDPCVNKVSQWLDMKVSYTHNLEKNLAALEKTMKELKAKRDDLERRLKRE
A4          MGNCVALEISCDQTLNHACGCLFGDRNYILKMEANLEALQNTMQELEERRDDLLRRVVIE
            **  :::.*  *  :*:..  *  . .*  ::* ** **::**:**: :**** **:  *

A3          EARGLQRLSEFQVWLDSVATVEDIIITLLRDRNVEIQRLCLCRFCSKSLTRSYRYGKSVF
A4          EDKGLQRLAQVQGWLSRVKDVCSQVNDLLKAKSIQTERLCLCGYCSKNFISGRNYGINVL
            * :*****::.* **. *  * . :  **: :.:: :***** :***.:  . .** .*:

A3          LRLREVEKLK-GEVFGVITEQASTSAFEERPLQPTIVGLDAMVGRAWNSLMKDER
A4          KKLKHVEGLLAKGVFEVVAEKIPAPKVEKKHI-QTTVGQDTMLDKAGKHLMEDG
             :*:.** *    ** *::*:  :  .*::  :  * ** *:*: :* : **:*


A5          MG--ISFSIPFDPCVNKVSQWLDMKVSYTHNLEKNLAALEKTMKELKAKRDDLERRLKRE
A6          MGNCVALEISCDQTLNHACGCLFGDRNYILKMEANLEALQNTMQELEERRDDLLRRVVIE
            **  :::.*  *  :*:..  *  . .*  ::* ** **::**:**: :**** **:  *

A5          EARGLQRLSEFQVWLDSVATVEDIIITLLRDRNVEIQRLCLCRFCSKSLTRSYRYGKSVF
A6          EDKGLQRLAQVQGWLSRVKDVCSQVNDLLKAKSIQTERLCLCGYCSKNFISGRNYGINVL
            * :*****::.* **. *  * . :  **: :.:: :***** :***.:  . .** .*:

A5          LRLREVEKLK-GEVFGVITEQASTSAFE
A6          KKLKHVEGLLAKGVFEVVAEKIPAPKVE
             :*:.** *    ** *::*:  :  .*
```

```
A7          MG--ISFSIPFDPCVNKVSQWLDMKVSYTHNLEKNLAALEKTMKELKAKRDDLERRLKRE
A8          MGNCVALEISCDQTLNHACGCLFGDRNYILKMEANLEALQNTMQELEERRDDLLRRVVIE
            **  :::.*  *  :*:..  *   . .*  ::* ** **::**:**: :**** **:  *

A7          EARGLQRLSEFQVWLDSVATVEDIIITLLRDRNVEIQRLCLCRFCSKSLTRSYRYGKSVF
A8          EDKGLQRLAQVQGWLSRVKDVCSQVNDLLKAKSIQTERLCLCGYCSKNFISGRNYGINVL
            * :*****::.* **. *  * . :  **: :.:: :***** :***.:  . .** .*:

A7          LRLREVEKLK-GEVFGVITEQASTSKVEKKHI-QTTVGLDAMVGRAWNSLMKDER
A8          KKLKHVEGLLAKGVFEVVAEKIPAPAFEERPLQPTIVGQDTMLDKAGKHLMEDG
             :*:.** *     ** *::*:   :  .*::  :  * ** *:*: :* : **:*


A9          MG--ISFSIPFDPCVNKVSQWLDMKVSYTHNLEKNLAALEKTMKELKAKRDDLERRLKRE
A10         MGNCVALEISCDQTLNHACGCLFGDRNYILKMEANLEALQNTMQELEERRDDLLRRVVIE
            **  :::.*  *  :*:..  *   . .*  ::* ** **::**:**: :**** **:  *

A9          EARGLQRLSEFQVWLDSVATVEDIIITLLRDRNVEIQRLCLCRFCSKSLTRSYRYGKSVF
A10         EDKGLQRLAQVQGWLSRVKDVCSQVNDLLKAKSIQTERLCLCGYCSKNFISGRNYGINVL
            * :*****::.* **. *  * . :  **: :.:: :***** :***.:  . .** .*:

A9          LRLREVEKLK-GEVFGVITEQASTSKVEKKHI-QTTVGQDTMLDKAGKHLMEDG
A10         KKLKHVEGLLAKGVFEVVAEKIPAPAFEERPLQPTIVGLDAMVGRAWNSLMKDER
             :*:.** *     ** *::*:   :  .*::  :  * ** *:*: :* : **:*
```

AT1G62630

```
MGISFSIPFDPCVNKVSQWLDMKGSYTHNLEKNLVALETTMEELKAKRDDLLRRLKREEDRGLQRLSEFQVWLNRVATVEDIIITLLRD
RDVEIQRLCLCRFCSKNLTTSYRYGKSVFLRLREVEKLKGEVFGVITEQASTSAFEERPLQPTIVGQKKMLDKAWKHLMEDGT
```

AT4G14610

```
MGGCISVSVSCDQFVNQFSQWLCVRKGYIHSLPENLAALQKAIEVLKTKHDDVKRRVDKEEFLGRRHRLSQVQVWLTNVVIIEKRFNDL
FSNKEVEIERLCFCGFCSKSFGKSYHYGKMVSVMLKEVENLSSRGVFDVVTEENLVAQVEEMPIQSTVVGQETMLERVWNTLMKDGF
```

```
B1          --MGISFSIPFDPCVNKVSQWLDMKGSYTHNLEKNLVALETTMEELKAKRDDLLRRLKRE
B2          MGGCISVSVSCDQFVNQFSQWLCVRKGYIHSLPENLAALQKAIEVLKTKHDDVKRRVDKE
                **.*:  *  **:.****  ::  .* *.*  :**.**:.::* **:*:**:  **:.:*

B1          EDRG-LQRLSEFQVWLNRVATVEDIIITLLRDRDVEIQRLCLCRFCSKNLTTSYRYGKSV
B2          EFLGRRHRLSQVQVWLTNVVIIEKRFNDLFSNKEVEIERLCFCGFCSKSFGKSYHYGKMV
            *  *  :***:.****..*.  :*.  :   *:  :::***:***:*  ****.:  .**:*** *

B1          FLRLREVEKLSSRGVFDVVTEENLVAQVEEMPIQSTVVGQETMLERVWNTLMKDGF
B2          SVMLKEVENLKG-EVFGVITEQASTSAFEERPLQPTIVGQKKMLDKAWKHLMEDGT
             :  *:***:*..   ** *:**:   .:  **  *:* *:***:.**::.*:  **:**
```

```
B3      --MGISFSIPFDPCVNKVSQWLDMKGSYTHNLEKNLVALETTMEELKAKRDDLLRRLKRE
B4      MGGCISVSVSCDQFVNQFSQWLCVRKGYIHSLPENLAALQKAIEVLKTKHDDVKRRVDKE
            **.*:  *  **:.**** :: .* *.* :**.**:.::* **:*:**: **:.:*

B3      EDRG-LQRLSEFQVWLNRVATVEDIIITLLRDRDVEIQRLCLCRFCSKNLTTSYRYGKSV
B4      EFLGRRHRLSQVQVWLTNVVIIEKRFNDLFSNKEVEIERLCFCGFCSKSFGKSYHYGKMV
        *  *  :***:.****..*. :*. :  *: :::***:***:* ****.: .**:*** *

B3      FLRLREVEKLKG-EVFGVITEQASTSAFEERPLQPTIVGQETMLERVWNTLMKDGF
B4      SVMLKEVENLSSRGVFDVVTEENLVAQVEEMPIQSTVVGQKKMLDKAWKHLMEDGT
         : *:***:*..  ** *:**:  .: .** *:* *:***:.**::.*: **:**


B5      --MGISFSIPFDPCVNKVSQWLDMKGSYTHNLEKNLVALETTMEELKAKRDDLLRRLKRE
B6      MGGCISVSVSCDQFVNQFSQWLCVRKGYIHSLPENLAALQKAIEVLKTKHDDVKRRVDKE
            **.*:  *  **:.**** :: .* *.* :**.**:.::* **:*:**: **:.:*

B5      EDRG-LQRLSEFQVWLNRVATVEDIIITLLRDRDVEIQRLCLCRFCSKNLTTSYRYGKSV
B6      EFLGRRHRLSQVQVWLTNVVIIEKRFNDLFSNKEVEIERLCFCGFCSKSFGKSYHYGKMV
        *  *  :***:.****..*. :*. :  *: :::***:***:* ****.: .**:*** *

B5      FLRLREVEKLKG-EVFGVITEQASTSAF
B6      SVMLKEVENLSSRGVFDVVTEENLVAQV
         : *:***:*..  ** *:**:  .: .


B7      --MGISFSIPFDPCVNKVSQWLDMKGSYTHNLEKNLVALETTMEELKAKRDDLLRRLKRE
B8      MGGCISVSVSCDQFVNQFSQWLCVRKGYIHSLPENLAALQKAIEVLKTKHDDVKRRVDKE
            **.*:  *  **:.**** :: .* *.* :**.**:.::* **:*:**: **:.:*

B7      EDRG-LQRLSEFQVWLNRVATVEDIIITLLRDRDVEIQRLCLCRFCSKNLTTSYRYGKSV
B8      EFLGRRHRLSQVQVWLTNVVIIEKRFNDLFSNKEVEIERLCFCGFCSKSFGKSYHYGKMV
        *  *  :***:.****..*. :*. :  *: :::***:***:* ****.: .**:*** *

B7      FLRLREVEKLKG-EVFGVITEQASTSAFEEMPIQSTVVGQETMLERVWNTLMKDGF
B8      SVMLKEVENLSSRGVFDVVTEENLVAQVEERPLQPTIVGQKKMLDKAWKHLMEDGT
         : *:***:*..  ** *:**:  .: .** *:* *:***:.**::.*: **:**


B9      --MGISFSIPFDPCVNKVSQWLDMKGSYTHNLEKNLVALETTMEELKAKRDDLLRRLKRE
B10     MGGCISVSVSCDQFVNQFSQWLCVRKGYIHSLPENLAALQKAIEVLKTKHDDVKRRVDKE
            **.*:  *  **:.**** :: .* *.* :**.**:.::* **:*:**: **:.:*

B9      EDRG-LQRLSEFQVWLNRVATVEDIIITLLRDRDVEIQRLCLCRFCSKNLTTSYRYGKSV
B10     EFLGRRHRLSQVQVWLTNVVIIEKRFNDLFSNKEVEIERLCFCGFCSKSFGKSYHYGKMV
        *  *  :***:.****..*. :*. :  *: :::***:***:* ****.: .**:*** *

B9      FLRLREVEKLKG-EVFGVITEQASTSQVEEMPIQSTVVGQKKMLDKAWKHLMEDGT
B10     SVMLKEVENLSSRGVFDVVTEENLVAAFEERPLQPTIVGQETMLERVWNTLMKDGF
         : *:***:*..  ** *:**:  .: .** *:* *:***:.**::.*: **:**
```

II. Swap mutants involving the N-terminus

At3G07040

MASATVDFGIGRILSVLENETLLLSGVHGEIDKMKKELLIMKSFLEDTHKHGGNGSTTTTTQLFQTFVANTRDLAYQIEDILDEFGYHI
HGYRSCAKIWRAFHFPRYMWARHSIAQKLGMVNVMIQSISDSMKRYYHSENYQAALLPPIDDGDAKWVNNISESSLFFSENSLVGIDAP
KGKLIGRLLSPEPQR

AT3G46710

MVDAITEFVVGKIDNYLIEEAPMLIGVKDDLEELKTELTCIQVYLKNVEVCDKEDEVSKEWTKLVLDIAYDVEDVLDTYFLKLEKRLHR
LGLMRLTNIISDKKDAYNILDDIKTLKRRTLDVTRKLEMYGIGNFNEHRVVASTSRVREVRRARSDDQEERVVGLTDDAKVLLTKLLDD
DGDNKI

```
7040PPL37 MVDAITEFVVGKIDNYLIEEAPMLIGVKDDLEELKTELLIMKSFLEDTHKHGGNGSTTTT
6710PPL38 MASATVDFGIGRILSVLENETLLLSGVHGEIDKMKKELTCIQVYLKNVEVCDKED-----
          *..* .:* :*:* . * :*: :* **: :::::*.**  :: :*::..    :

7040PPL37 TQLFQTFVANTRDLAYQIEDILDEFGYHIHGYRSCAKIWRAFHFPRYMWARHSIAQKLGM
6710PPL38 -EVSKEWTKLVLDIAYDVEDVLDTYFLKLEKRLHRLGL---------MRLTNIISDKKDA
           :: : :.  . *:**::**:** :  ::.       :         *   . *::*

7040PPL37 VNVMI------Q---SISDSMKRYYHSENYQAALLPPIDDGDAKWVNNISESSLFFSENS
6710PPL38 YNILDDIKTLKRRTLDVTRKL-EMYGIGNFNEHRVVA----STSRVREVRRARSDDQEER
           *::       :   .:: .: . *   *::   :      .:. *.:: .:    .*:

7040PPL37 LVGIDAPKGKLIGRLLSPEP--QR
6710PPL38 VVGLTDDAKVLLTKLLDDDGDNKI
          :**:      *: :**. :   :
```

III. Point mutations

AT1G12290(WT)
MGGCVSVQVSCDQLLNHLGRCFCRKLYYIQNIKENLTSLEEAMEDLKALRDDLLRKVQTAEEGGLQRLHQIKVWLKRVKTI
ESQFNDLDSSRTVELQRLCCCGVGSRNLRLSYDYGRRVFLMLNIVEDLKSKGIFEEVAHPATRAVGEERPLQPTIVGQETI
LEKAWDHLMDDGT

M1
MGGCVSVQVSCDQLLNHLGRCFCRKLYYIQNIKENLTSLEEAMEDLKALRDDLLRKVQTAEEGGLQRLHQIKVWLKRVKTI
ESQFNDLDSSRTVELQRLCCCGVGSRNLRLSYDYGRRVFLMLNIVEDLKSKGIFEEVAHPATRAVGEERPLQPTIVGQETI
LEKAWDHAMDDGT

M2
MGGCVSVQVSCDQLLNHLGRCFCRKLYYIQNIKENLTSLEEAMEDLKALRDDLLRKVQTAEEGGLQRLHQIKVWLKRVKTI
ESQFNDLDSSRTVELQRLCCCGVGSRNLRLSYDYGRRVFLMLNIVEDLKSKGIFEEVAHPATRAVGEERPLQPTIVAQETI
LEKAWDHLMDDGT

M3
MGGCVSVQVSCDQLLNHLGRCFCRKLYYIQNIKENLTSLEEAMEDLKALRDDLLRKVQTAEEGGLQRLHQIKVWLKRVKTI
ESQFNDLDSSRTVELQRLCCCGVGSRNLRLSYDYGRRVFLMLNIVEDLKSKGIFEEVAHPATRAVGAARPLQPTIVGQETI
LEKAWDHLMDDGT

M4
MGGCVSVQVSCDQLLNHLGRCFCRKLYYIQNIKENLTSLEEAMEDLKALRDDLLRKVQTAEEGGLQRLHQIKVWLKRVKTI
ESQFNDLDSSRTVELQRLCCCGVGSRNLRLSYDYGRRVFLMLNIVEDLKSKGIFEEVAHPATRAVGKKRPLQPTIVGQETI
LEKAWDHLMDDGT
