# Supplementary material for: Genome-wide functional analyses of plant coiled–coil NLR-type pathogen receptors reveal essential roles of their N-terminal domain in oligomerization, networking, and immunity
Source: PLoS Biol. 2018 Dec 12;16(12):e2005821. doi: 10.1371/journal.pbio.2005821 (PMC6312357; doi:10.1371/journal.pbio.2005821)
Supplement: S1 Fig — Residues common to frequent interactors (Fig 2B) are highlighted in dark blue. The first letter preceding the name of each gene indicates its corresponding Group. Hydrophobic amino acids within CC domains are highlighted in yellow, and A and D heptad positions are displayed in bold. At-Col-0, Arabidopsis thaliana ecotype Columbia-0; CC, coiled–coil; CN, CC–NB; CNL, CC–NLR. (PDF) [file pbio.2005821.s001.pdf]

Yellow highlight - hydrophobic amino acids
orange - highly conserved Trp residue upstream motif in CC-domains
purple/red highlight - regular / atypical residues 'EDVID' motif
dark blue highlight - the four amino acids in Group D
black highlight - charged motif within linker
green highlight - 'VG' and p-loop
red font - leucine in pre-p-loop region
bold - heptad positions A and D

```
Heptads:      -------AbcDefgAbcDefgAb---cDefgAbcDefg....AbcDefgAbcDefgAbcDefg.----..........AbcDefgAbcDefgAbcDefgAbcDefg--------.....AbcDe---fgAbcDefgAbcD...
MLA           -------MDIVTGAISNLIPKLG---ELLTEEFKLHKGVKKNIEDLGKELESMNAALIKIGEVP----REQLDSQDKLWADEVRELSYVIEDVVDKFLVQVDGIQSD--------DNNNKFKGLM---KRTTELLKKVKHKHGIAHA--------IKDIQEQLQ-KVADRRDRNKVFV--PHPTRTIAIDPCLRALYA-----EATELVGIYGKRDQGLMRLLSMEGDDASNKRLKKVSIVGFGGLGKTTLA
Sr33          ---------SNAGAIAKLIPKLG---ELLVGEYKLHKGVKKNIEDLLKELKTMNAALIKIGEVP----PDQLDSQDKLWADEVRELSYVIEDAVDKFLVRVHGVEPD--------DNTNGFKGLM---KRTTKLLKKVVDKHGIAHA--------IKDIKKELQ-EVAARRDRNKFDG--IASIPTEAIDPRLRALYI-----EAAELVGIYGKRDQELMSLLSLEGDDASTKKLKKVSIVGFGGLGKTTLA
Rx            -------------MAYAAVTSLM---RTIHQSMELTGCD---LQPFYEKLKSLRAILEKSCNI------MGDHEGLTILEVEIVEVAYTTEDMVDSESRNVFLAQNLEERSRAMWEIFFVLEQAL---ECIDSTVKQWMATSDSMKD-----------------------------LK-PQ-TSSLVSLPEHDVEQ------PENIMVG--RENEFEMMLDQLARGG----RELEVVSIVGMGGIGKTTLA

D AT1G58848   ---------MAGELISFGIQNLW---NLLSQECELFQGVEDQVTELKRDLNLLSSFLKDADAK------KHTSAVVKNCVEEIKEIIYDGEDTIETFVLEQNLGKTS----GIK----KSIRRLA---CIIPDRRRYALGIGGLSNR--------ISKVIRDMQ-SFGVQQAIVDGGYKQPQ-G-D-KQREMRPRFSKD----DDSDFVG--LEANVKKLVGYLVDEA-----NVQVVSITGMGGLGKTTLA
D AT1G59218   ---------MAGELISFGIQNLW---NLLSQECELFQGVEDQVTELKRDLNLLSSFLKDADAK------KHTSAVVKNCVEEIKEIIYDGEDTIETFVLEQNLGKTS----GIK----KSIRRLA---CIIPDRRRYALGIGGLSNR--------ISKVIRDMQ-SFGVQQAIVDGGYKQPQ-G-D-KQREMRPRFSKD----DDSDFVG--LEANVKKLVGYLVDEA-----NVQVVSITGMGGLGKTTLA
D AT1G59124   ---------MAGELISFGIQNLW---NLLSQECELFQGVEDQVTELKRDLNMLSSFLKDANAK------KHTSAVVKNCVEEIKEIIYDGEDTIETFVLEQNLGKTS----GIK----KSIRRLA---CIIPDRRRYALGIGGLSNR--------ISKVIRDMQ-SFGVQQAIVDGGYKQPQ-G-D-KQREMRQKFSKD----DDSDFVG--LEANVKKLVGYLVDEA-----NVQVVSITGMGGLGKTTLA
D AT1G58807   ---------MAGELISFGIQNLW---NLLSQECELFQGVEDQVTELKRDLNMLSSFLKDANAK------KHTSAVVKNCVEEIKEIIYDGEDTIETFVLEQNLGKTS----GIK----KSIRRLA---CIIPDRRRYALGIGGLSNR--------ISKVIRDMQ-SFGVQQAIVDGGYKQPQ-G-D-KQREMRQKFSKD----DDSDFVG--LEANVKKLVGYLVDEA-----NVQVVSITGMGGLGKTTLA
D AT1G58390   ---------MAGELVSFGIKKLW---DLLSQECEQFQGVEDQVTGLKRDLNLLSSFLKDADAK------KHTTAVVRNVVEEIKEIVYDAEDIIETYLLKEKLWKTS----GIK----MRIRRHA---CIISDRRRNALDVGGIRTR--------ISDVIRDMQ-SFGVQQAIVDGGYMQPQ-G-D-RQREMRQTFSKD----YESDFVG--LEVNVKKLVGYLVDEE-----NVQVVSITGMGGLGKTTLA
D AT1G59780   ----MQDLYMVDSIVSFGVEKLW---KLLSQEYERFQGVEEQITELRDDLKMLMAFLSDADAK------KQTRALARNCLEEIKEITYDAEDIIEIFLLK-----------GS----VNMRSLA---CFPGGRREIALQITSISKR--------ISKVIQVMQ-NLGIKSDIMDGVDSHA--Q-LERKRELRHTFSSE----SESNLVG--LEKNVEKLVEELVGND-----SSHGVSITGLGGLGKTTLA
D AT1G58602   ---------MAGELVSFAVNKLW---DLLSHEYTLFQGVEDQVAELKSDLNLLKSFLKDADAK------KHTSALVRYCVEEIKDIVYDAEDVLETFVQKEKLGTTS----GIR----KHIKRLT---CIVPDRREIALYIGHVSKR--------ITRVIRDMQ-SFGVQQMIVDDYMHPL--R-N-REREIRRTFPKD----NESGFVA--LEENVKKLVGYFVEED-----NYQVVSITGMGGLGKTTLA
D AT1G58400   ---------MVEAIVSFGVEKLW---DRLTQEYEQFQGVEDRIAELKSNLNLLKSFLKDAEAK------KNTSQMVRHCVEEIKEIVYDTENMIETFILKEAARKRS----GII----RRITKLT---CIKVHRWEFASDIGGISKR--------ISKVIQDMH-SFGVQQMISDGSQSSHLLQ-E-REREMRQTFSRG----YESDFVG--LEVNVKKLVGYLVEED-----DIQIVSVTGMGGLGKTTLA
D AT1G58410   ----------MELVSFGVEKLW---DRLSQEYDQFKGVEDQVTELKSNLNLLKSFLKDADAK------KHISEMVRHCVEEIKDIVYDTEDIIETFILKEKVEMKR----GIM----KRIKRFA---STIMDRRELASDIGGISKR--------ISKVIQDMQ-SFGVQQIITDGSRSSHPLQ-E-RQREMRHTFSRD----SENDFVG--MEANVKKLVGYLVEKD-----DYQIVSLTGMGGLGKTTLA
D AT5G43470   ---------MAEAFVSFGLEKLW---DLLSRESERLQGIDGQLDGLKRQLRSLQSLLKDADAK------KHGSDRVRNFLEDVKDLVFDAEDIIESYVLNKLSGKGK----GVK----KHVRRLA---CFLTDRHKVASDIEGITKR--------ISEVIGEMQ-SFGIQQIIDGGRSLSLQER-QRVQREIRQTYPDS----SESDLVG--VEQSVKELVGHLVEND-----VHQVVSIAGMGGIGKTTLA
D AT5G48620   ---------MAEGFVSFGLEKLW---DLLSRESERLQGIDEQLDGLKRQLRSLQSLLKDADAK------KHGSDRVRNFLEDVKDLVFDAEDIIESYVLNKLRGEGK----GVK----KHVRRLA---RFLTDRHKVASDIEGITKR--------ISDVIGEMQ-SFGIQQIIDGVRSLSLQER-QRVQREIRQTYPDS----SESDLVG--VEQSVEELVGHLVEND-----IYQVVSIAGMGGIGKTTLA
D AT5G35450   ---------MAEGVVSFGVQKLW---ALLNRESERLNGIDEQVDGLKRQLRGLQSLLKDADAK------KHGSDRVRNFLEDVKDLVFDAEDIIESYVLNKLRGEGK----GVK----NHVRRLA---CFLTDRHKVASDIEGITKR--------ISKVIGEMQ-SLGIQQQIIDGGRSLSL-Q-D-IQREIRQTFPNS----SESDLVG--VEQSVEELVGPMVEID-----NIQVVSISGMGGIGKTTLA
D AT1G53350   ---------MAEAVVSFGVEKLW---ELLSRESARLNGIDEQVDGLKRQLGRLQSLLKDADAK------KNETERVRNFLEDVKDIVYDADDIIESFLLNELRGKEK----GIK----KQVRTLA---CFLVDRRKFASDIEGITKR--------ISEVIVGMQ-SLGIQHIADGGGRSLSL-Q-E-RQREIRQTFSRN----SESDLVG--LDQSVEELVDHLVEND-----SVQVVSVSGMGGIGKTTLA
D AT1G59620   ---------MAETLLSFGVEKLW---DLLVRESDRFQGVKKQFNELRSDLNKLRCFLEDADAK------KHQSAMVSNTVKEVKEIVYDTEDIIETFLRKKQLGRTR----GMK----KRIKEFA---CVLPDRRKIAIDMEGLSKR--------IAKVICDMQ-S--------------------------------------LGV--QQENVKKLVGHLVEVE----DSSQVVSITGMGGIGKTTLA

C AT1G50180   ---------MAEAIVSVTVQKLG---QLLLEEPLFLFGIGDQVKQLQDELKRLNCFLKDADEK------QHESERVRNWVAGIREASYDAEDILEAFFLKAESRKQK-GMKRVL----RRLACIL---NEAVSLHSVGSEIREITSR--------LSKIAASML-DFGIKESMGRE--GLSL---SDSLREQRQSFPYV----VEHNLVG--LEQSLEKLVNDLVSGG----EKLRVTSICGMGGLGKTTLA
C AT3G46730   ---------MVDAVTGFVLNKIG---GYLINEVLALMGVKDDLEELKTELTCIHGYLKDVEAR------EREDEVSKEWTKLVLDIAYDIEDVLDTYFLKLEERSLR----RGL----LRLTNKI---GKKRDAYNIVEDIRTLKRR--------ILDITRKRE-TFGIGSFNEPRG-ENIT---NVRVRQLRRAPPVD----QEELVVG--LEDDVKILLVKLLSDNEK--DKSYIISIFGMGGLGKTALA
C AT3G46530   ---------MVDAITEFVVGKIG---NYLIEEASMFMAVKEDLEELKTELTCIHGYLKDVEAR------EREDEVSKEWSKLVLDFAYDVEDVLDTYHLKLEERSQR----RGL----RRLTNKI---GRKMDAYSIVDDIRILKRR--------ILDITRKRE-TYGIGGLKEPQGGGNTS---SLRVRQLRRARSVD----QEEVVVG--LEDDAKILLEKLLDYE-E--KNRFIISIFGMGGLGKTALA
C AT3G46710   ---------MVDAITEFVVGKID---NYLIEEAPMLIGVKDDLEELKTELTCIQVYLKNVEVC------DKEDEVSKEWTKLVLDIAYDVEDVLDTYFLKLEKRLHR----LGL----MRLTNII---SDKKDAYNILDDIKTLKRR--------TLDVTRKLE-MYGIGNFNEHR--VVAS---TSRVREVRRARSDD----QEERVVG--LTDDAKVLLTKLLDDDGD--NKIYMISIFGMEGLGKTSLA
C AT3G14470   --MTGIGEMFLAAFLQALFQTLVS--EPFRSFFKRRELNENLLERLSTALLTITAVLIDAEEK------QITNPVVEKWVNELRDVVYHAEDALDDIATEALRLNIG-AESSSSNRLRQLRGRMSLGDFLDGNSEHLETRLEKVTIR--------LERLASQRN-ILGLKELTAMIPKQ--------RLPTT----SLV----DESEVFG--RDDDKDEIMRFLIPENGKD-NGITVVAIVGIGGVGKTTLS
C AT3G14460   -----MANSYLSSCANVMVERINT--SQELVELCKGKSSSALLKRLKVALVTANPVLADADQR------AEHVREVKHWLTGIKDAFFQAEDILDELQTEALRRVV-AEAGGLG---GLFQNLM--AGREAIQKKIEPKMEKVVRL--------LEHHVKHIE-VIGLKEYSETREPQ--------WRQASRSRPDDL----PQGRLVG--RVEDKLALVNLLLSDDEISIGKPAVISVVGMPGVGKTTLT
C AT3G50950   ---------MVDAVVTVFLEKTL---NILEEKGRTVSDYRKQLEDLQSELKYMQSFLKDAERQ------KRTNETLRTLVADLRELVYEAEDILVDCQLADGDDGNE-QRSSNA----WLSRLHP---ARVPLQYKKSKRLQEINER--------ITKIKSQVE-PYFEFITPSNV--------GRDNGTDRWSSPVY----DHTQVVG--LEGDKRKIKEWLFRSN-D--SQLLIMAFVGMGGLGKTTIA
C AT3G07040   ---------MASATVDFGIGRIL---SVLENETLLLSGVHGEIDKMKKELLIMKSFLEDTHKHGGNGSTTTTTQLFQTFVANTRDLAYQIEDILDEFGYHIHGYRSC----AKI----WRAFHFP---RYMWARHSIAQKLGMVNVM--------IQSISDSMK-RYYHSENYQAALLPPIDDGDAKWVNNISESSLFF----SENSLVG--IDAPKGKLIGRLLSPE---PQRIVVAVVGMGGSGKTTLS

B AT1G12290   MGGCVSVQVSCDQLLNHLGRCFCRKLYYIQNIKENLTSLEEAMEDLKALRDDLLRKVQTAEEG-----GLQRLHQIKVWLKRVKTIESQFNDLDSSR-----TVELQ-----------RLCCC-GVGSRNLRLSYDYGRRVFLMLNI--------VEDLKSKG--IFEEVAHPATR---------A------VGEERP-----LQPTIVG--QETILEKAWDHLMDDG-----T-KIMGLYGMGGVGKTTLL
B AT4G10780   MGSCISLQISCDQVLTRAYSCFFSLGNYIHKLKDNIVALEKAIEDLTATRDDVLRRVQMEEGK-----GLERLQQVQVWLKRVEIIRNQFYDLLSAR-----NIEIQ-----------RLCFY-SNCSTNLSSSYTYGQRVFLMIKE--------VENLNSNG--FFEIVAAPAPK------------------LEMRP-----IQPTIMG--RETIFQRAWNRLMDDG-----V-GTMGLYGMGGVGKTTLL
B AT5G47250   ---MNCCWQVVEPCYKSALSYLCVKVGNICMLKENLVLLKSAFDELKAEKEDVVNRVNAGELK-----GGQRLAIVATWLSQVEIIEENTKQLMDVASARDASSQNA-----------SAVRRRLSTSGCWFSTCNLGEKVFKKLTE--------VKSLSGK---DFQEVTEQPPP---------P------VVEVRL-----CQQT-VG--LDTTLEKTWESLRK---------DMLGIFGMGGVGKTTLL
B AT4G26090   MDFISSLIVG-CAQVLCESMNMAERRGHKTDLRQAITDLETAIGDLKAIRDDLTLRIQQDGLE-----GRSCSNRAREWLSAVQVTETKTALLLVRF-----RREQ-----------RTRMRRRYLSCFGCADYKLCKKVSAILKS--------IGELRERS--EAIKTDG-GSI---------Q------VTCREI-----PIKSVVG--NTTMMEQVLEFLSEEE-----ERGIIGVYGPGGVGKTTLM
B AT1G12220   MGGCFSVSLPCDQVVSQFSQLLCVRGSYIHNLSKNLASLQKAMRMLKARQYDVIRRLETEEFT----GRQQRLSQVQVWLTSVLIIQNQFNDLLRSN-----EVELQ-----------RLCLC-GFCSKDLKLSYRYGKRVIMMLKE--------VESLSSQG--FFDVVSEATPF---------A------DVDEIP-----FQPTIVG--QEIMLEKAWNRLMEDG-----S-GILGLYGMGGVGKTTLL
B AT1G12280   MGACLTLSFSCDEVVNQISQGLCINVGYICELSKNVVAMKKDMEVLKKKRDDVKRRVDIEEFT----RRRERLSQVQGWLTNVSTVENKFNELLTTN-----DAELQ-----------RLCLF-GFCSKNVKMSYLYGKRVVLMLKE--------IESLSSQG--DFDTVTLATPI---------A------RIEEMP-----IQPTIVG--QETMLERVWTRLTEDG-----D-EIVGLYGMGGVGKTTLL
B AT1G12210   MGGCVSVSLSCDREVNQFSQWLCVSGSYIQNLSENLASLQKAMGVLNAKRDDVQGRINREEFT----GHRRRLAQVQVWLTRIQTIENQFNDLLSTC-----NAEIQ-----------RLCLC-GFCSKNVKMSYLYGKRVIVLLRE--------VEGLSSQG--VFDIVTEAAPI---------A------EVEELP-----IQSTIVG--QDSMLDKVWNCLMEDK-----V-WIVGLYGMGGVGKTTLL
B AT1G62630   M--GISFSIPFDPCVNKVSQWLDMKGSYTHNLEKNLVALETTMEELKAKRDDLLRRLKREEDR-----GLQRLSEFQVWLNRVATVEDIIITLLRDR-----DVEIQ-----------RLCLC-RFCSKNLTTSYRYGKSVFLRLRE--------VEKLKGE---VFGVITEQAST---------S------AFEERP-----LQPTIVG--QKKMLDKAWKHLMEDG-----T-GIMGMYGMGGVGKTTLL
B AT1G63360   M--GISFSIPFDPCVNKVSQWLDMKVSYTHNLEKNLAALEKTMKELKAKRDDLERRLKREEAR-----GLQRLSEFQVWLDSVATVEDIIITLLRDR-----NVEIQ-----------RLCLC-RFCSKSLTRSYRYGKSVFLRLRE--------VEKLKGE---VFGVITEQAST---------S------AFEERP-----LQPTIVG--QDTMLDKAGKHLMEDG-----V-GIMGMYGMGGVGKTTLL
B AT1G63350   M--GISFSIPFDPCVNKVSQWLDMKVSYTHNLEKNLVALETTMEELKAKRDDLLRKLKREEDR-----GLQTLGEIKVWLNRVETIESRVNDLLNAR-----NAELQ-----------RLCLC-GFCSKSLTTSYRYGKSVFLKLRE--------VEKLERR---VFEVISDQAST---------S------EVEEQQ-----LQPTIVG--QETMLDNAWNHLMEDG-----V-GIMGLYGMGGVGKTTLL
B AT5G47260   MGNNFSVESPSLAPFLCGKRK------YLYNLERNLEALHKVMQDLNAMRNDLLKRLSKEEEI-----GLQGLQEVKEWISMVEEIEPKANRLLDES-----VSEIQ-----------RLSRY-GYCSLIPASTYRYSEKVLTTMEG--------VETLRSKG--VFEAVVHRALP---------P------LVIKMP-----PIQLTVS--QAKLLDTAWARLMDIN-----V-GTLGIYGRGGVGKTTLL
B AT5G05400   MGACFSVAISCDQAVNNLTSCLSRNQNRFRNLVDHVAALKKTVRQLEARRDDLLKRIKVQEDR-----GLNLLDEVQQWLSEVESRVCEAHDILSQS-----DEEID-----------NLCCG-QYCSKRCKYSYDYSKSVINKLQD--------VENLLSKG--VFDEVAQKGPI---------P------KVEERL-----FHQEIVG--QEAIVESTWNSMMEVG-----V-GLLGIYGMGGVGKTTLL
B AT1G15890   MGNCVALEISCDQTLNHACGCLFGDRNYILKMEANLEALQNTMQELEERRDDLLRRVVIEEDK-----GLQRLAQVQGWLSRVKDVCSQVNDLLKAK-----SIQTE-----------RLCLC-GYCSKNFISGRNYGINVLKKLKH--------VEGLLAKG--VFEVVAEKIPA---------P------KVEKKH-----IQ-TTVG--LDAMVGRAWNSLMKDE-----R-RTLGLYGMGGVGKTTLL
B AT5G63020   MGGCVSVSISCDQLTKNVCSCLNRNGDYIHGLEENLTALQRALEQIEQRREDLLRKILSEERR-----GLQRLSVVQGWVSKVEAIVPRVNELVRMR-----SVQVQ-----------RLCLC-GFCSKNLVSSYRYGKRVMKMIEE--------VEVLRYQG--DFAVVAERVDA---------A------RVEERP-----TR-PMVA--MDPMLESAWNRLMEDE-----I-GILGLHGMGGVGKTTLL
B AT5G43740   M--LGWLVIPWNQIFTAACGCFLSDRNYIHMMESNLDALQKTMEELKNGRDDLLGRVSIEEDK-----GLQRLAQVNGWLSRVQIVESEFKDLLEAM-----SIETG-----------RLCLL-GYCSEDCISSYNYGEKVSKMLEE--------VKELLSKK--DFRMVAQE-II---------H------KVEKKL-----IQTT-VG--LDKLVEMAWSSLMNDE-----I-GTLGLYGMGGVGKTTLL
B AT5G43730   M-VDWLSLLPWNKIFTAACGCFLSDSNYIHLMESNLDALQKTMEELKNGRDDLLARVSIEEDK-----GLQRLALVNGWLSRVQIVESEFKDLLEAM-----SIETG-----------RLCLF-GYCSEDCISSYNYGGKVMKNLEE--------VKELLSKK--NFEVVAQK-II---------P------KAEKKH-----IQTT-VG--LDTMVGIAWESLIDDE-----I-RTLGLYGMGGIGKTTLL
B AT1G51480   M-ADWLLLIPWNKIFTAACGCFFSDRNYIHKMEANLDDLHTTMEELKNGRDDLLRRVSIEEDK-----GLQQLAQVKGWISRVEIVESRFKDLLEDK-----STETG-----------RLCLF-GFCSENCISSYNYGEKVMKNLEE--------VKELLSKK--HFEVVAHKIPV---------P------KVEEKN-----IHTT-VG--LYAMVEMAWKSLMNDE-----I-RTLCLHGMGGVGKTTLL
B AT1G61180   MGSCFSLQVS--DQTLNRIFNCLIGKSYIRTLEKNLRALQREMEDLRAIQHEVQNKVARDEAR-----HQRRLEAVQVWLDRVNSVDIECKDLLSVT-----PVELQ-----------KLCLC-GLCSKYVCSSYKYGKKVFLLLEE--------VKKLNSEG--NFDEVSQ-PPP---------R------SEVEER-----PTQPTIG--QEDMLEKAWNRLMEDG-----V-GIMGLHGMGGVGKTTLF
B AT1G61310   MGSCFSFQIAVGDQTMNRIFDCLIGKSYIRTLEQNLRALQREMEDLRATQHEVQNKVAREESR-----HQQRLEAVQVWLDRVNSIDIECKDLLSVS-----PVELQ-----------KLCLC-GLCTKYVCSSYKYGKKVFLLLEE--------VKILKSEG--NFDEVSQ-PPP---------R------SEVEER-----PTQPTIG--QEEMLEKAWNRLMEDG-----V-GIMGLHGMGGVGKTTLF
B AT1G61190   MGNFVCIEISG-DQMLDRIIRCLCGKGYIRNLEKNLRALQREMEDLRATQHEVQNKVAREESR-----HQQRLEAVQVWLDRVNSIDIECKDLLSVS-----PVELQ-----------KLCLC-GLCSKYVCSSYKYGKRVFLLLEE--------VTKLKSEG--NFDEVSQ-PPP---------R------SEVEER-----PTQPTIG--QEEMLKKAWNRLMEDG-----V-GIMGLHGMGGVGKTTLF
B AT4G27190   ---MECCAPVIGEILRLMYESTFSRVANAIKFKSNVKALNESLERLTELKGNMSEDHETLLTK-----DKPLRLKLMRWQREAEEVISKARLKL-----------EER-----------VSC--------GMSLRPRMSRKLVKILDE--------VKMLEKDGIEFVDMLSVESTP---------E------RVEHVP-----GVSVVHQTMASNMLAKIRDGLTSEK-----A-QKIGVWGMGGVGKTTLV
B AT4G14610   MGGCISVSVSCDQFVNQFSQWLCVRKGYIHSLPENLAALQKAIEVLKTKHDDVKRRVDKEEFL----GRRHRLSQVQVWLTNVVIIEKRFNDLFSNK-----EVEIE-----------RLCFC-GFCSKSFGKSYHYGKMVSVMLKE--------VENLSSRG--VFDVVTEENLV---------A------QVEEMP-----IQSTVVG--QETMLERVWNTLMKDG-----F-KIMGLYGMGGVGKTT--

A ATT5G6690   --MNDWASLGIGSIGEAVFSKLL---KVVIDEAKKFKAFKPLSKDLVSTMEILFPLTQKIDSM-----QKEL-DFGVKELKELRDTIERADVAVRKFPR---VKWYE-KSKYTRK--IERINKDMLKFCQIDLQLLQHRNQLTLLGLT-GNLVNSVDGLSKRMD-LLSVPAPV--F-----------RDLCSVPKLD--------KVIVG--LDWPLGELKKRLLD------DSVVTLVVSAPPGCGKTTLV
A AT5G66910   MVVVDWLGLGLGSVAGALVSEGL---KVLISEAKKVLAFKSVSNELASTMESLLPVIKEIESM-----QDG------MELQDLKDTIDKALLLVEKCSHVEKWNIIL-KSKYTRK--VEEINRKMLKFCQVQLQLLLFRNQLKSMPSMEAILNNYFQNINKKLD-RLSGSPAPPLV-----------SKRCSVPKLDN--------MVLVG--LDWPLVELKKKLLD--------NSVVVVSGPPGCGKTTLV
A AT5G66630   MPISDVASLVGGAALGAPLSEIF---KLVIEEAKKVKDFKPLSQDLASTMERLVPIFNEIDMM-----QQGS-NRGTSELKVLTETMERAGEMVHKCSRIQWYSIAK-KALYTRE--IKAINQDFLKFCQIELQLIQHRNQLQYMRSM-G-----MASVSTKAD-LLSDIGNE--F-----------SKLCLVAQPEV--------VTKFW--LKRPLMELKKMLFE------DGVVTVVVSAPYALGKTTLV
A AT4G33300   --M-AITDFFAGEIATELLKQLF---TISTTAWRY----KNTAKQLLTLIDSIRPTIKEIQYS-----GVELPAHRQAQIGMLFDTLEKGKKLTDKVLSSKRWNLYR-QLTLARK--MEKLEKTISNFLKNEVFTHILADVHHLRAD--------TSVRLDRVD-MSLDRVIQQVGSMKIGGGGLISEAMKRAEAMEIETNDDSEKFGVG--LELGKVKVKKMMFE------SQGGVFGISGMGGVGKTTLA
A AT5G04720   -----MADIIGGEVVTELVRQLY---AVSQKTLRC----RGIAKNLATMIDGLQPTIKEIQYS-----GVELTPHRQAQLRMFSETLDKCRKLTEKVLKSSRWNMVR-QLLHVRK--MENLQSKVSSFLNGQLLVHVLADVHHVRAD--------SEFRFDRID-RKVDSLNEKLGSMKLRGSESLREALKTAEATVEMVTTDGADLGVG--LDLGKRKVKEMLFKSI---DGERLIGISGMSGSGKTTLA
A AT1G33560   --MASFIDLFAGDITTQLLKLLA---LVANTVYSC----KGIAERLITMIRDVQPTIREIQYS-----GAELSNHHQTQLGVFYEILEKARKLCEKVLRCNRWNLKH-VY-HANK--MKDLEKQISRFLNSQILLFVLAEVCHLRVN--------GDRIERNMD-RLLTERNDSLSF--------PETMMEIETVSDPE---IQTVLELG--K-KKVKEMMFKFTD--------THLFGISGMSGSGKTTLA

E AT1G10920   -------------------------------------------------------------------------------------------------------------------------------------------------MK-SLGIQEIIDGASSM--------SLQERQREQ-KEIRQTFANS------------------------------------SESDLVG--VEQSVEALAGHLVEND-----NIQVVSISGMGGIGKTTLA
E AT3G15700   -------------MGKDFKSMVTRCIYVGKENDNVKKLKTATEELKDLRNIVMKRVKMYEDQ---QKLKRLEKVQVWLRQADVAIKEAEEMLI-----------T-----------LMSSS-SSNGSSMMSFHKLDKKLCKKLKE--------VQEIKSRG--TFDVVVENSGI---------G--SGSMMISNVD-----RDDQTVG--LEAVSGLVWRCMTVDN-----T-GIIGLYGVEGVGKTTVL
E AT1G52660   -------------MGKDFKSLVTRCIYVGKMNDNAKKLKIATEELKDLGNNVMKRVKLCEEQ---QQMKRLDKVQTWLRQADTVIKEAEEYF------------------------LMSSS-SSSSGLISSSHKMEKKICKKLKE--------VQEIKSRG--MFEVVAESTGG---------IGGGAGGGLTIKD-----SDEQTIG--LEAVSGLVWRCLTMEN-----T-GIIGLYGVEGVGKTTVL
E AT4G27220   ----------------------------MFRSNARALNRALERLKNVQTKVNEALKRSGIQ-----EKSLERKLRIWLRKVEENVPLGELILE-----------KR-----------SSCAI-----WLSDKDVEILEKVKRLEEQ--------GQDLIKKI--SVNKSSREIVE-----------------RVLGPS-----FHPQKTA---LEMLDKLKDCLKKKN-----V-QKIGVWGMGGVGKTTLV
E AT1G61300   --------------------------------------------------------------------------------------------------------------------------------------------------MGCCFSVQFSFDDQTLV--------RIFNFLCGNINRNSFGVEER-----------------------------------PTQPTIG--QEEMLEKAWNRLME-D-----RVGIMGLHGMGGVGKTTLF
E AT4G19060   -----------MDIAKKFISEID---DKLESKSEFDKELEKIKSSFNEEYEKWSSGKQRGSSSKHGNQSTHGDSSPTRNSSGSSKKGRPKANRVET-----------------------------------SSELPDHLIRGFINEKLF---------LKNFL------------------------------------------------LKQKESEEF-----------KTLAIVGKYGVGKTTLC
E AT5G45440   ---------------------MT---QEDSSRGLTSVGRVDFTNRFADRYNEW--------------LGTTG----------DETKQV---EDRVET-----------------------------------DSGLPGHDIYGFENEIKS---------LQHFL------------------------------------------------LDQKSYKLF-----------KSLVVVGEYGVGKTALC
E AT5G45490   ---------------------MP---SKNLQQ---AVLTNEFTTNFITTCKDW--------------LDVNGLAKGNLEKKRDDNEE---EERLKT-----------------------------------ESKLPGHDIHGFDNEIKS---------LQHFL------------------------------------------------LDQKVRREF-----------KSLVIVGEYGVGKTALC
```
